# Molecular basis of mucopolysaccharidosis IVA (Morquio A syndrome): A review and classification of *GALNS* gene variants and reporting of 68 novel variants

**DOI:** 10.1002/humu.24270

**Published:** 2021-08-23

**Authors:** Alessandra Zanetti, Francesca D'Avanzo, Moeenaldeen AlSayed, Ana Carolina Brusius‐Facchin, Yin‐Hsiu Chien, Roberto Giugliani, Emanuela Izzo, David C. Kasper, Hsiang‐Yu Lin, Shuan‐Pei Lin, Laura Pollard, Akashdeep Singh, Rodolfo Tonin, Tim Wood, Amelia Morrone, Rosella Tomanin

**Affiliations:** ^1^ Laboratory of Diagnosis and Therapy of Lysosomal Disorders, Department of Women's and Children's Health University of Padova Padova Italy; ^2^ Fondazione Istituto di Ricerca Pediatrica Città della Speranza Padova Italy; ^3^ King Faisal Specialist Hospital and Research Centre, Faculty of Medicine Alfaisal University Riyadh Saudi Arabia; ^4^ Medical Genetics Service/HCPA, DR BRASIL Research Group/HCPA, and INAGEMP Porto Alegre Brazil; ^5^ Department of Medical Genetics and Pediatrics National Taiwan University Hospital Taipei Taiwan; ^6^ Department of Genetics/UFRGS Medical Genetics Service/HCPA, DR BRASIL Research Group/HCPA, and INAGEMP Porto Alegre Brazil; ^7^ BioMarin Pharmaceutical Inc. Novato California USA; ^8^ ARCHIMED Life Science GmbH Vienna Austria; ^9^ Division of Genetics and Metabolism, Departments of Pediatrics and Medical Research MacKay Memorial Hospital Taipei Taiwan; ^10^ Biochemical Diagnostic Laboratory Greenwood Genetic Center Greenwood South Carolina USA; ^11^ Molecular and Cell Biology Laboratory, Pediatric Neurology Unit and Laboratories Meyer Children's Hospital Florence Italy; ^12^ Department of Neurosciences, Psychology, Pharmacology and Child Health University of Florence Florence Italy

**Keywords:** *GALNS*, lysosomal storage disorder, Morquio A syndrome, MPS IVA, mucopolysaccharidosis IVA, N‐acetylgalactosamine‐6‐sulfate

## Abstract

Mucopolysaccharidosis IVA (MPS IVA, Morquio A syndrome) is a rare autosomal recessive lysosomal storage disorder caused by mutations in the N‐acetylgalactosamine‐6‐sulfatase (*GALNS*) gene. We collected, analyzed, and uniformly summarized all published *GALNS* gene variants, thus updating the previous mutation review (published in 2014). In addition, new variants were communicated by seven reference laboratories in Europe, the Middle East, Latin America, Asia, and the United States. All data were analyzed to determine common alleles, geographic distribution, level of homozygosity, and genotype‐phenotype correlation. Moreover, variants were classified according to their pathogenicity as suggested by ACMG. Including those previously published, we assembled 446 unique variants, among which 68 were novel, from 1190 subjects (including newborn screening positive subjects). Variants' distribution was missense (65.0%), followed by nonsense (8.1%), splicing (7.2%), small frameshift deletions(del)/insertions(ins) (7.0%), intronic (4.0%), and large del/ins and complex rearrangements (3.8%). Half (50.4%) of the subjects were homozygous, 37.1% were compound heterozygous, and 10.7% had only one variant detected. The novel variants underwent in silico analysis to evaluate their pathogenicity. All variants were submitted to ClinVar (https://www.ncbi.nlm.nih.gov/clinvar/) to make them publicly available. Mutation updates are essential for the correct molecular diagnoses, genetic counseling, prenatal and preimplantation diagnosis, and disease management.

## Graphical abstract

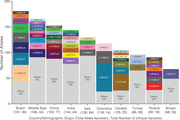

In this mutation update, we collected, analyzed, classified and uniformly summarized all published GALNS gene variants. In addition, 68 new variants were communicated by seven reference laboratories in Europe, the Middle East, Latin America, Asia, and the United States.

## 1 INTRODUCTION

Mucopolysaccharidosis IVA (MPS IVA or Morquio A syndrome; MIM# 253000) is an autosomal recessive lysosomal storage disorder caused by mutations in the *GALNS* gene, which encodes for the enzyme N‐acetylgalactosamine‐6‐sulfatase (GALNS; EC 3.1.6.4). Importantly, reduced or totally absent GALNS enzyme activity leads to impaired degradation of the glycosaminoglycans (GAGs) chondroitin‐6‐sulfate (C6S) and keratan sulfate (KS) and their subsequent accumulation in tissues (Khan et al., 2017; Matalon et al., 1974). C6S and KS are mainly produced in cartilage and are stored primarily in the lysosomes and extracellular matrix of this tissue, leading to skeletal and connective tissue abnormalities (Khan et al., 2017; Morrone, Caciotti, et al., 2014).

MPS IVA is a rare disease, with an estimated prevalence varying from 1 in 71,000 births in the United Arab Emirates, to 1 in 323,000 births in Denmark, and to 1 in 1,872,000 births in Malaysia (Leadley et al., 2014). The clinical presentation of MPS IVA disease shows a spectrum of phenotypes ranging from a classical, rapidly progressing early‐onset form to a nonclassical, slowly progressing, late‐onset form. An intermediate slowly progressing form with early‐onset has also been identified (Lee et al., 2012; Tüysüz et al., 2019). The classical disease phenotype typically presents in the first year of life with systemic bone dysplasia, short trunk dwarfism, spinal cord compression, cervical instability, joint laxity, pulmonary compromise, abdominal hernia, and corneal opacification (Galimberti et al., 2018; Hendriksz et al., 2013; Lin et al., 2014; Peracha et al., 2018). If untreated, these symptoms lead to death typically in the second decade (Lavery & Hendriksz, 2015; Lin et al., 2020). In nonclassical forms, symptoms may not appear or be recognized until later in childhood, or even until early adulthood (Galimberti et al., 2018; Montaño et al., 2007; Tüysüz et al., 2019) and may include minor skeletal abnormalities, such as a less pronounced short stature (Moisan et al., 2020). Patients with milder forms also have a longer life expectancy (Prat et al., 2008; Sawamoto et al., 2020).

Unfortunately, due to the rarity of the disease, the difficult differential diagnosis, and the clinical heterogeneity (Peracha et al., 2018), it may take months or even years from symptom onset to the diagnosis (Galimberti et al., 2018; Hendriksz et al., 2013; Rigoldi et al., 2018). Enzyme replacement therapy (ERT) with recombinant human GALNS (elosulfase alpha) is currently the only approved disease‐specific treatment option for patients with MPS IVA (Hendriksz et al., 2015) and can improve endurance, respiratory function, and quality of life (Hendriksz et al., 2014; Hendriksz et al., 2016, 2018). Moreover, early intervention with ERT may improve bone growth (Akyol et al., 2019). Thus, timely diagnosis and intervention may optimize treatment outcomes and reduce mortality.

The classical diagnostic approach starts with suspicion of MPS IVA, often based on clinical signs and skeletal radiographs. With the introduction of pilot or routine newborn screening (NBS) programs, presymptomatic neonates can also precociously come to light, due to low GALNS enzymatic activity in dried blood spots (Chien et al., 2020; Lin et al., 2020). In both approaches, MPS IVA diagnosis is confirmed by the enzyme assay of GALNS activity in leukocytes or fibroblasts (Hendriksz et al., 2013; Peracha et al., 2018) followed by molecular analysis.

Standard DNA sequencing is routinely used as the first‐level molecular analysis to detect causative variants in *GALNS* exons and in their flanking sequences, thus allowing confirmation of diagnoses based on biochemical analyses and aiding in genetic counseling (Filocamo et al., 2018). With advances in next‐generation sequencing (NGS) and the availability of gene panels for groups of diseases or symptoms, molecular diagnosis may sometimes precede enzyme testing in the diagnostic pathway. However, in some cases, both Sanger sequencing and NGS approaches might fail to identify the pathogenic alleles (Caciotti et al., 2018). In a few cases, additional approaches are implemented to verify the presence of genetic alterations commonly not detected by first‐level analyses (i.e., genetic rearrangements, deep intronic alterations, etc.) (Caciotti et al., 2015, 2018).

The *GALNS* gene (Ensembl ID: ENSG00000141012), located on chromosome 16q24.3, contains 14 exons and is approximately 43 kb in length (Ensembl, 2020). A previous review of MPS IVA variants identified 277 published unique alterations, most of them being missense variants distributed throughout the coding sequence and at flanking splice sites (Morrone, Caciotti, et al., 2014). Introns containing Alu repetitive elements can result in recombination events, potentially leading to large deletions (up to 8.0 kb) and/or rearrangements (Hori et al., 1995). In this study, we uniformly collected and summarized all published *GALNS* gene variants, updating the previous mutation review (Morrone, Caciotti, et al., 2014). In addition, previously undescribed genotypes were communicated by seven reference laboratories in Europe, the Middle East, Latin America, Asia, and the United States. When possible, data were analyzed to determine the most common alleles, geographic distribution, levels of homozygosity, and genotype‐phenotype correlation. Variants were classified according to their pathogenicity as suggested by the American College of Medical Genetics and Genomics and the Association for Molecular Pathology (ACMG/AMP) (Richards et al., 2015). A summary of all *GALNS* variants so far identified will aid in the interpretation of molecular results and help to confirm the diagnosis in patients with suspected MPS IVA. Including those previously published, we collected 446 unique variants, among which 68 were novel, from a total of 1190 subjects. Novel variants were further analyzed by in silico tools to predict their potential pathogenicity to aid clinical interpretation.

## 2 METHODS

### 2.1 Editorial policies and ethical considerations

All participants (or parents/guardians) who provided samples for genetic testing in this study gave their informed consent for biochemical and molecular analyses.

### 2.2 Literature search and collection of novel variants

A literature search was performed in PubMed and Google using the search terms “*GALNS* variants” and “*GALNS* mutations.” Publications were filtered from the last *GALNS* mutation update (Morrone, Caciotti, et al., 2014) to December 2020. Additional variants were retrieved from the Human Gene Mutation Database (HGMD) Professional 2020.1. Results were limited to studies in humans. Each publication meeting the search criteria was screened by two reviewers for information regarding *GALNS* variants. Reports of individuals with *GALNS* variants, where variants were reported as linked to MPS IVA, were extracted and assessed to reduce redundancy where possible. Data related to the different degrees of disease severity were reported as indicated in each publication.

Unpublished data of subjects with a clinical diagnosis of MPS IVA and/or low/absent GALNS enzymatic activity, as well as neonates who tested positive for MPS IVA in NBS, were reported by diagnostic laboratories and clinical units participating in the present project.

### 2.3 Sequencing

Details regarding DNA extraction, target amplification/enrichment, Sanger sequencing, and NGS are available in the supplementary materials. Primer sequences will be made available upon request.

### 2.4 Variant annotation and correction of misreported variants

Variants were annotated according to the guidelines of the Human Genome Variation Society (HGVS) nomenclature, version 20.05 (den Dunnen et al., 2016). For the description of sequence variants, we used reference sequence NM_000512.5 for the *GALNS* gene and the corresponding protein sequence NP_000503.1. Validation of variant annotations was performed by Name Checker (https://mutalyzer.nl/name-checker) for exonic variants and by Variant Validator (https://variantvalidator.org/) for intronic variants. Large deletions and complex rearrangements were checked manually and, when possible, annotated on chromosome 16 using NC_000016.10 as a reference sequence (GRCh38.p13 genome build). When discrepancies were found, corrections were made accordingly. All misreported variants and other discrepancies were recorded.

### 2.5 In silico prediction of novel variants' pathogenicity

In silico analyses to determine the novel variants' pathogenicity were performed by ANNOVAR (version 08‐06‐2020), using the table_annovar.pl program with dbnsfp41a and dbscsnv11 as filter‐based annotation databases (https://annovar.openbioinformatics.org/) (K. Wang et al., 2010). For these analyses, the pathogenicity scores of the following tools were considered: SIFT, FATHMM, MutationAssessor, PolyPhen2, MutationTaster, PROVEAN, GERP++, CADD, REVEL, and dbscSNV. The analysis was considered performed if at least one tool provided an output. Small and large deletions/insertions and intronic variants, except for variants on canonical splice sites, were not analyzable by this approach.

### 2.6 Variants' classification according to ACMG/AMP guidelines and ClinVar submission

Clinical classification of each variant according to ACMG/AMP recommendations (Richards et al., 2015) was performed. Further supporting information was collected from the literature and from public databases (gnomAD, dbSNPs, ClinVar, and UniProt). Supporting evidence included enzyme activity in homozygous subjects and/or results of in vitro functional studies, results of in silico analyses, subjects' ethnicity, parents' consanguinity, allele frequency, and enzyme structure. During the classification process, the recommendations of the Sequence Variant Interpretation Working Group of the ClinGen initiative were followed, when possible, to correctly apply the ACMG/AMP criteria (https://clinicalgenome.org/working-groups/sequence-variant-interpretation/). Specific application of each ACMG/AMP criterion to MPS IVA and the *GALNS* gene are reported in Table S1. All variants were then submitted to the ClinVar database with associated evidence and literature references to make them publicly available (ClinVar accession numbers: SCV001547566‐SCV001548006).

## 3 VARIANTS

### 3.1 *GALNS* mutation spectrum

Overall, 42 articles were evaluated; 40 publications since 2014 were found from PubMed and Google searches. Of these, 38 were articles and two were published abstracts. Two additional articles (L. Wang et al., 1999; Whybra et al., 2012), published before 2014, that were not included in the previous update were recovered by comparison of our list of variants with that reported by HGMD Professional 2020.1. Moreover, two other articles (Erazo‐Narváez et al., 2020; Ficicioglu et al., 2020) were recently published and, owing to the timing of the present article submission, were not included in the whole analysis. Ficicioglu et al. (2020) reported three African American siblings who were compound heterozygotes for c.611A>G [p.(Asn204Thr)] and c.964G>C [p.(Ala322Pro)]. Erazo‐Narváez et al. (2020) reported two Colombian women carrying the variants c.491A>C [p.(Asn164Thr)] and c.901G>T [p.(Gly301Cys)]. All of these variants had been previously described, with the exception of c.964G>C, which has been included in the present list of *GALNS* unique variants (Table S2).

Overall, including those previously published, 446 unique variants were collected from 1190 genotyped subjects. The current update provides an additional 169 unique variants to the 277 previously reported (Table S2). Five of the 169 unique variants were reported only at the protein level (nucleotide alterations were not provided in the reporting papers). Annotation checks of variants revealed several misreported variants that were corrected accordingly, when possible (Table S3).

Of the 654 genotypes identified in the current update, 448 (68.5%) were collected from the literature and 206 (31.5%) were reported by communications from laboratories (Table 1). On the whole, 53 patients were related to family members with the same variant; 48 were sibling pairs, two patients were twins, and one group of three patients were siblings. In addition, the genotypes of 43 neonates (13 from literature and 30 from laboratories) that tested positive at the NBS were collected. All of these ultimately resulted in the aggregation of 2323 alleles for which at least one alteration of the *GALNS* gene was reported, from 1190 individuals diagnosed with MPS IVA or who tested positive to the NBS (Tables S4 and S5). The total count of 2323 alleles also comprises 82 gene alterations reported in the original papers as tabular lists without information about their occurrence in patients (Morrone, Caciotti, et al., 2014). Due to this lack of information, these instances were not included in the list of genotypes.

**Table 1:** Summary of collected genotypes

	Number of genotypes		
	2014 update (Morrone, Caciotti, et al., 2014)	2020 update (this study)	Total
Genotypes from literature	536	448	984
Genotypes from laboratory communications	0	206	206
Total	536	654	1190^a^

^a^ Total number of collected genotypes (*n* = 1190) includes the 43 genotypes from subjects positive to newborn screening, 13 from the literature, and 30 from laboratory communications.

**Figure 1:**
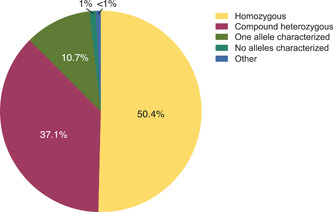
Distribution of subjects' zygosity. Percentage distribution of zygosity of the collected genotypes (*n* = 1190). “Other” refers to subjects in which >2 variants were described

Of the identified individuals, 600 (50.4%) were homozygous for *GALNS* variants, 442 (37.1%) were compound heterozygous, 127 (10.7%) had only one allele characterized, and 12 (1.0%) individuals no alleles characterized (Figure 1). As for these last two categories of patients, original publications did not mention the performing of additional analyses to search for large deletions/duplications and/or gross rearrangements (Bochernitsan et al., 2018; Jezela‐Stanek et al., 2019; Leong et al., 2019; Szklanny et al., 2018; Tapiero‐Rodriguez et al., 2018; Tüysüz et al., 2019). Notably, three subjects with two different homozygous variants were described previously (Bunge et al., 1997; Tomatsu, Filocamo, et al., 2004) and in this study. Six subjects (two of whom were siblings) carrying three distinct variants were reported previously (Cozma et al., 2015; Morrone, Tylee, et al., 2014; Tulebayeva et al., 2020), and in this study; segregation analysis was not available in any of these cases.

Subjects varied considerably with respect to national/geographic origin and ethnic backgrounds. Sex was reported for 968 of 1190 (81.3%) individuals, showing a distribution of 45.9% females and 54.1% males.

**Figure 2:**
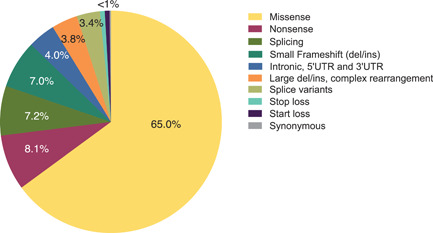
Distribution of unique variant types (*n* = 446)

In agreement with the previous report (Morrone, Caciotti, et al., 2014), most unique variants here reported were missense (65.0%), followed by nonsense (8.1%), splice site variants (7.2%), and small frameshift deletions or insertions (7.0%). All other variant types each had a frequency ≤4% (Figure 2). At the time of the previous update, few large deletions or insertions had been reported, potentially due to underdetection (Morrone, Caciotti, et al., 2014). Here, we report 15 subjects with 17 unique large deletions, insertions, or complex rearrangements.

### 3.2 Most frequently reported *GALNS* alleles

**Figure 3:**
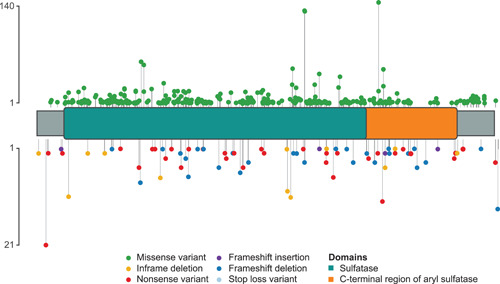
Schematic representation of the distribution of exonic variants in the GALNS protein. Exonic variants, excluding large deletions, insertions, and complex rearrangements, are represented in a schematic of the GALNS protein (522 amino acids). Missense variants are reported at the top of the figure and the remaining exonic variants at the bottom. “Lollipop” lengths represent variant frequency

Commonly reported variants occurred throughout the length of the *GALNS* gene with no particular “hotspot” regions for variation (Figure 3).

The 10 most commonly reported variants collectively occurred in 625 alleles and accounted for only 26.9% of all alleles (Table 2), demonstrating the heterogeneity of alterations in the *GALNS* gene from subjects with MPS IVA. Nine of these 10 alleles were missense and 1 was a splice variant.

**Table 2:** Most frequently^a^ described *GALNS* alleles by reported ethnicity/country

Alleles by country/ethnicity	Number detected	Percentage of that allele's total	Percentage of all detected alleles
c.1156C>T [p.(Arg386Cys)]	140	100.0	6.0
Colombian	27	19.3	1.2
Brazilian	19	13.6	0.8
Italian	13	9.3	0.6
Polish	12	8.6	0.5
Spanish	11	7.9	0.5
Argentinian	7	5.0	0.3
Chinese	5	3.6	0.2
Chilean	3	2.1	0.1
Turkish	3	2.1	0.1
Not available/not reported	20	14.3	0.9
All other countries^b^	20	14.3	0.9
c.901G>T [p.(Gly301Cys)]	127	100.0	5.5
Colombian	55	43.3	2.4
Brazilian	24	18.9	1.0
Canadian	13	10.2	0.6
Spanish	9	7.1	0.4
Portuguese	8	6.3	0.3
French	3	2.4	0.1
Not available/not reported	4	3.1	0.2
All other countries^b^	11	8.7	0.5
c.337A>T [p.(Ile113Phe)]	57	100.0	2.5
Irish	27	47.4	1.2
British	15	26.3	0.6
British/Irish	3	5.3	0.1
Other	3	5.3	0.1
Not available/not reported	6	10.5	0.3
All other countries^b^	3	5.3	0.1
c.346G>A [p.(Gly116Ser)]	53	100.0	2.3
Brazilian	20	37.7	0.9
Arab	16	30.2	0.7
Turkish	3	5.7	0.1
Not available/not reported	2	3.8	0.1
All other countries^b^	12	22.6	0.5
c.120+1G>A[‐]	49	100.0	2.1
Tunisian	43	87.8	1.9
Brazilian	3	6.1	0.1
Not available/not reported	3	6.1	0.1
c.860C>T [p.(Ser287Leu)]	48	100.0	2.1
Indian	12	25.0	0.5
Greek	4	8.3	0.2
Middle Eastern	4	8.3	0.2
Turkish	4	8.3	0.2
Not available/not reported	9	18.8	0.4
All other countries^b^	15	31.3	0.6
c.1171A>G [p.(Met391Val)]	47	100.0	2.0
Canadian	32	68.1	1.4
French	4	8.5	0.2
Not available/not reported	6	12.8	0.3
All other countries^b^	5	10.6	0.2
c.953T>G [p.(Met318Arg)]	39	100.0	1.7
Chinese	19	48.7	0.8
Taiwanese	13	33.3	0.6
All other countries^b^	7	17.9	0.3
c.1023C>G [p.(Ser341Arg)]	37	100.0	1.6
Brazilian	33	89.2	1.4
All other countries^b^	4	10.8	0.2
c.415G>A [p.(Gly139Ser)]	28	100.0	1.2
Asian‐multiethnic	4	14.3	0.2
Not available/not reported	9	32.1	0.4
All other countries^b^	15	53.6	0.6

*Note:* NCBI reference sequences: NM_000512.5 for the *GALNS* gene and NP_000503.1 for the GALNS protein.

^a^ Only alleles described ≥3 times are reported.

^b^ Includes all other countries with allele frequency <3.

The alleles most frequently reported were the missense changes c.1156C>T [p.(Arg386Cys)] and c.901G>T [p.(Gly301Cys)] most often in Colombia, and c.337A>T [p.(Ile113Phe)], most common in people of Irish descent (Table 2). These three alleles were also the most frequent in both the 2014 update (Morrone, Caciotti, et al., 2014) and in the 2005 update (Tomatsu et al., 2005). c.120+1G>A and c.29G>A [p.(Trp10*)] were the most commonly described splicing and nonsense alleles respectively. Notably, many *GALNS* alleles were only reported once (149 alleles, 33.4%) or twice (106 alleles, 23.8%).

### 3.3 Geographic distribution of *GALNS* alleles

**Figure 4:**
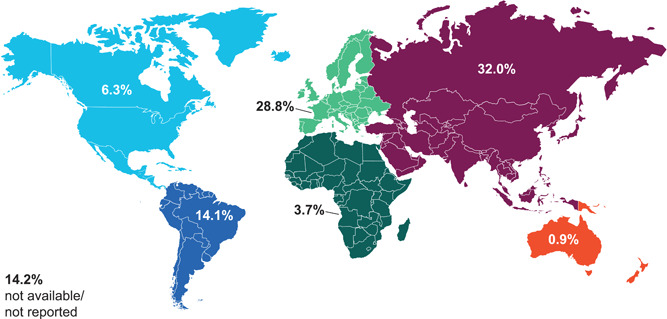
Distribution of reported *GALNS* alleles by continent

**Figure 5:**
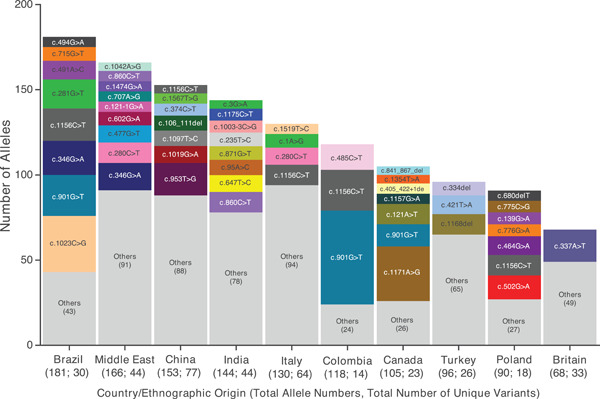
Most common alleles† for the 10 most frequent nationalities/ethnic backgrounds reported. †Alleles registered ≥5 times are reported. “Others” are variants reported <5 times. Numbers related to “others” are shown within parentheses. Total allele numbers include those alleles for which originating countries/ethnographic origins were available/reported. Total number of unique variants is shown after the semicolon on the *x*‐axis. Middle East includes Saudi Arabia, Iraq, Iran, and Oman. NCBI reference sequences: NM_000512.5 for the *GALNS* gene and NP_000503.1 for the GALNS protein

Geographical information was available for 1024 individuals (86.1%), with subjects originating from all continents (Figure 4). Allelic heterogeneity was apparent among all individual populations. However, if we consider the ratio between the number of unique variants reported in a specific population and the total number of alleles described for the same population (Figure 5), individuals from China (77:153) and Italy (64:130) were the most heterogeneous, whereas those from Brazil (30:181) and Colombia (14:118) were the least heterogeneous.

Brazil and the Middle East were the most highly represented, contributing 7.8% and 7.1% of all alleles, respectively. Variant c.1023C>G [p.(Ser341Arg)] was the most commonly reported among Brazilian individuals, accounting for 18.2% of the alleles described in this population. Previously, this variant was also reported in Brazilian subjects and only in two other subjects from Sri Lanka, with heterozygous status (Tomatsu, Dieter, et al., 2004) and was suggested to be a founder mutation (Bochernitsan et al., 2018). In agreement with this, in the present update, 89.2% of all c.1023C>G alleles were reported in Brazil. Variant c.901G>T [p.(Gly301Cys)] was the next most represented allele in Brazil (13.3% of all Brazilian alleles) and also accounted for 46.6% and 12.4% of alleles reported in Colombia and Canada, respectively (Moisan et al., 2020; Moreno Giraldo et al., 2018; Tapiero‐Rodriguez et al., 2018).

Variant c.1156C>T [p.(Arg386Cys)] was the most widely distributed allele in the top 10 represented countries, appearing in Colombia, Brazil, Italy, Poland, and Spain, overall representing 3.5% of all identified alleles. Variant c.485C>T [p.(Ser162Phe)], previously reported to account for 12.9% of alleles in Colombia (Tapiero‐Rodriguez et al., 2018) was the only other variant reported ≥5 times in Colombia (12.7% of Colombian alleles).

In Canada, the most commonly reported variant, considered to be a French‐Canadian founder variant (Moisan et al., 2020), was c.1171A>G [p.(Met391Val)], which accounted for 30.5% of reported Canadian alleles. The variants with the largest proportion of alleles in the Middle East were c.346G>A [p.(Gly116Ser)], c.280C>T [p.(Arg94Cys)], and c.477G>T [p.(Trp159Cys)], accounting for 9.6%, 7.2%, and 6.0% of alleles, respectively, in this region. In addition, variants c.602G>A [p.(Gly201Glu)], c.1474G>A (p.(Ala492Thr)], and c.1042A>G [p.(Thr348Ala)] were reported exclusively in the Middle East region. The most commonly reported variants in Turkey, c.1168del [p.(Leu390*)], c.421T>A [p.(Trp141Arg)], and c.334del [p.(Glu112Argfs*17)], accounted for 12.5%, 11.5% and 8.3%, respectively, of the *GALNS* alleles in the country and they have been described exclusively in Turkish families (Bunge et al., 1997; Khedhiri et al., 2014; Morrone, Tylee, et al., 2014; Terzioglu et al., 2002).

In alignment with the previous 2014 update (Morrone, Caciotti, et al., 2014), c.953T>G [p.(Met318Arg)] was the most commonly reported variant in China (12.4% of China's total), and alleles carrying this variant were described only in the South‐East Asian region. c.860C>T [p.(Ser287Leu)] was the most common allele in India (8.3% of Indian alleles), but was also reported in the Middle East (3.6% of all Middle Eastern alleles) and in several other countries worldwide. Among the most common Indian alleles, c.647T>C [p.(Phe216Ser)] has been reported to be rare in other populations, while c.95A>C [p.(Asn32Thr)] was reported only in alleles from Indian subjects (Bidchol et al., 2014).

The Italian population shared the c.280C>T [p.(Arg94Cys); 7.7% of Italian alleles] variant with the Middle Eastern population. Italy and Britain had the highest proportion of alleles reported <5 times among all other countries: 72.3% of all Italian alleles and 72.1% of all British alleles respectively. The only variant occurring >5 times in Britain (27.9% of British alleles) was c.337A>T [p.(lle113Phe)]. This variant was previously identified as common in subjects from the British Isles and in those of Irish descent (Morrone, Caciotti, et al., 2014). Variant c.502G>A [p.(Gly168Arg)] was the most commonly reported variant in Poland, followed by variant c.464G>A [p.(Gly155Glu)] (15.6% and 12.2% of all Polish alleles, respectively).

### 3.4 Newly identified variants

The genotypes of 206 subjects were collected from seven laboratories (Table S5), among which 68 novel genetic alterations were reported (Table 3). Ninety‐one individuals were homozygous, 82 were heterozygous, 30 had only one allele characterized, and for one subject both alleles remained unidentified. In addition, one patient presented three different variants and one patient presented two homozygous variants. Forty‐six out of 68 (67.6%) were missense variants, followed by small deletions (6), large deletions/complex rearrangements (5), nonsense (4), intronic/UTR (4), splicing (2), and stop‐loss (1). Variants' pathogenicity was evaluated through 10 selected in silico tools of ANNOVAR.

**Table 3:** Novel *GALNS* variants and evaluation of their pathogenicity

Nucleotide change	Predicted amino acid change	Number of alleles	Variant identifier	Predicted ACMG classification
c.‐42G>T	‐	3	‐	VUS
c.77dup	p.(Ala27Argfs*19)	1	‐	Likely pathogenic
c.121‐210C>T	‐	1	rs75552025	VUS
c.131G>T	p.(Gly44Val)	1	‐	VUS
c.142G>A	p.(Asp48Asn)	2	‐	VUS
c.239C>G	p.(Ser80Trp)	4	‐	VUS
c.245‐2A>G	‐	1	rs1352162269	Likely pathogenic
c.260T>C	p.(Leu87Pro)	1	‐	VUS
c.265G>T	p.(Gly89*)	1	‐	Likely pathogenic
c.268C>G	p.(Arg90Gly)	3	‐	VUS
c.274C>T	p.(Pro92Ser)	1	‐	VUS
c.296C>T	p.(Thr99Ile)	1	‐	VUS
c.313A>G	p.(Arg105Gly)	1	‐	VUS
c.319G>C	p.(Ala107Pro)	1	‐	VUS
c.323A>G	p.(Tyr108Cys)	1	rs1256041500	VUS
c.326C>T	p.(Thr109Ile)	2	‐	VUS
c.406A>C	p.(Lys136Gln)	1	rs750953060	VUS
c.491A>G	p.(Asn164Ser)	1	rs761725425	VUS
c.508T>C	p.(Tyr170His)	1	‐	VUS
c.529A>C	p.(Asn177His)	4	‐	VUS
c.563G>A	p.(Gly188Asp)	1	‐	VUS
c.567‐3C>T	‐	3	rs549597016	VUS
c.578A>G	p.(Glu193Gly)	1	rs1427663367	VUS
c.638C>T	p.(Ala213Val)	2	rs770239604	VUS
c.651_652insG	p.(Lys218Glufs*45)	2	rs1468285336	Pathogenic
c.664C>T	p.(Arg222Trp)	1	rs146963745	VUS
c.700G>A	p.(Ala234Thr)	1	rs368603508	VUS
c.706C>T	p.(His236Tyr)	1	rs1228027865	Likely pathogenic
c.707A>C	p.(His236Pro)	2	‐	Likely pathogenic
c.722C>A	p.(Ala241Asp)	4	‐	VUS
c.763G>A	p.(Gly255Arg)	3	rs1336648211	VUS
c.839_841del	p.(Asn280del)	1	rs1389218771	VUS
c.863del	p.(Asp288Alafs*31)	2	‐	Pathogenic
c.869G>A	p.(Gly290Asp)	1	‐	VUS
c.895C>T	p.(Gln299*)	1	‐	Likely pathogenic
c.(898+1_899‐1) _(1002+1_1003‐1)del	‐	2	‐	Pathogenic
c.(898+1_899‐1) _(1002+1_1003‐1)dup	‐	4	‐	VUS
c.899‐397_1003‐1862del	‐	4	‐	Pathogenic
c.909C>G	p.(Asn303Lys)	2	rs751446283	VUS
c.911G>T	p.(Gly304Val)	2	rs758439379	VUS
c.916T>G	p.(Phe306Val)	4	‐	VUS
c.917T>G	p.(Phe306Cys)	1	rs759590432	VUS
c.919C>A	p.(Leu307Met)	4	‐	VUS
c.934A>T	p.(Thr312Ser)	1	‐	VUS
c.940T>G	p.(Phe314Val)	1	rs774781295	VUS
c.943G>C	p.(Glu315Gln)	1	‐	VUS
c.956G>C	p.(Arg319Thr)	2	‐	VUS
c.985C>A	p.(His329Asn)	1	‐	VUS
c.1009del	p.(His337Thrfs*19)	1	‐	Likely pathogenic
c.1042A>G	p.(Thr348Ala)	5	‐	VUS
c.1142C>G	p.(Pro381Arg)	1	‐	VUS
c.1159G>A	p.(Gly387Ser)	1	‐	VUS
c.1164C>A	p.(Asp388Glu)	2	rs752339162	VUS
c.1192del	p.(His398Thrfs*43)	2	‐	Pathogenic
c.1196A>G	p.(Lys399Arg)	1	‐	VUS
c.1221C>G	p.(Asn407Lys)	2	‐	VUS
c.(1242+1_1243‐1)_(1364+1_1365‐1)del	‐	1	‐	Likely pathogenic
c.1261G>A	p.(Gly421Arg)	1	‐	VUS
c.(1364+1_1365‐1) _(1482+1_1483‐1)del	‐	2	‐	Pathogenic
c.1420C>T	p.(Gln474*)	2	‐	Pathogenic
c.1423C>A	p.(His475Asn)	1	rs749297663	VUS
c.1447C>T	p.(Gln483*)	1	‐	Likely pathogenic
c.1461C>A	p.(Asn487Lys)	1	‐	VUS
c.1483‐15A>G	‐	1	rs1461992033	VUS
c.1483‐2A>G	‐	1	‐	Likely pathogenic
c.1492C>T	p.(Pro498Ser)	1	rs1454253268	VUS
c.1558T>C	p.(Trp520Arg)	4	rs398123434	VUS
c.1567T>C	p.(*523Glnext*92)	1	‐	VUS

*Note:* NCBI reference sequences: NM_000512.5 for the *GALNS* gene and NP_000503.1 for the GALNS protein.

Abbreviations: ACMG, American College of Medical Genetics and Genomics; VUS, variant of uncertain significance.

This analysis was feasible for 54 of 68 (79.4%) novel variants (Table S7). For 35 variants, the analysis predicted a damaging effect, with almost all tools giving a score in the damaging range. For nine variants, the output was conflicting, but with a prevalence of damaging predictions. For nine other variants, the effect was not predictable given a comparable number of damaging and tolerated output scores. Finally, for one variant, a likely tolerated effect was predicted.

### 3.5 ACMG/AMP classification of *GALNS* variants

The ACMG/AMP classification of the 446 GALNS variants collected in this update revealed that for most variants, not enough evidence was available to classify their pathogenicity. Indeed, 259 variants (58.1%) were classified as variants of “uncertain significance.” The remaining variants could be classified as “likely pathogenic” (107; 24.0%), “pathogenic” (75; 16.8%), “likely benign” (1; 0.2%), or “benign” (4; 0.9%). The huge number of variants falling in the “uncertain significance” class is consistent with the high genetic heterogeneity shown by the *GALNS* gene and it is also strongly influenced by the absence of robust functional evidence for most variants (i.e., in vitro evaluation of enzymatic activity, in vivo activity for homozygotes, etc.).

All classified variants and their associated pathogenic evidence were submitted to ClinVar, where they can be retrieved by the following accession numbers: SCV001547566‐SCV001548006.

### 3.6 Homozygotes and genotype‐phenotype correlation

The genotype‐phenotype correlation was evaluated only in homozygous subjects to avoid the analysis being influenced by interference from the second allele. Overall, 600 of 1190 (50.4%) subjects were homozygotes, and clinical phenotype was reported for 314 (52.3%) of these. With reference to the genotypes collected following the 2014 update, 142 of 254 (55.9%) and 37 of 91 (40.7%) homozygous subjects were associated with clinical phenotypes within the literature or laboratory communications, respectively.

The 314 overall subjects analyzable for genotype‐phenotype correlation included 135 different variants (Table S6). Of these, 103 variants were associated with the classical phenotype, 19 were associated with a nonclassical phenotype, and two were associated with an intermediate clinical phenotype. Finally, eleven variants were reported with conflicting clinical phenotypes. Of these, seven were associated with either classical or nonclassical, two with either classical or intermediate, one with either intermediate or nonclassical, and one associated with all three phenotypes. The inconsistency of phenotypic classification for the same genotype may reflect the overall heterogeneity of the disease; in some cases, it may reflect the different ages at which diagnosis occurred in the subjects.

Variants associated with a clinical phenotype in ≥5 subjects are shown in Table 4. The majority of these variants resulted in classical MPS IVA form. The c.901G>T [p.(Gly301Cys)] variant was the most common variant associated with the classical phenotype (28/260 [10.8%] of subjects) and it was classified as nonclassical or intermediate in two subjects. Likewise, c.1171A>G [p.(Met391Val)] was the most common variant associated with the nonclassical phenotype (8/48 [16.7%] of subjects), with a different classification (classical) in one patient.

**Table 4:** Variants associated with a clinical phenotype in ≥5 homozygous subjects

Nucleotide change	Amino acid change	Clinical phenotype (number of homozygous subjects)			Total
		Classical	Intermediate	Nonclassical	
c.901G>T	p.(Gly301Cys)	28	1	1	30
c.1156C>T	p.(Arg386Cys)	16	1	‐	17
c.120+1G>A	‐	11	‐	‐	11
c.346G>A	p.(Gly116Ser)	9	‐	‐	9
c.1171A>G	p.(Met391Val)	1	‐	8	9
c.860C>T	p.(Ser287Leu)	7	‐	‐	7
c.139G>A	p.(Gly47Arg)	4	‐	1	5
c.280C>T	p.(Arg94Cys)	5	‐	‐	5
c.1019G>A	p.(Gly340Asp)	5	‐	‐	5
c.1168del	p.(Leu390*)	5	‐	‐	5

*Note:* NCBI reference sequences: NM_000512.5 for the *GALNS* gene and NP_000503.1 for the GALNS protein.

### 3.7 Variants detected by newborn screening programs

A total of 43 neonates identified through pilot NBS studies, evaluating GALNS enzyme activity in dried blood spots (DBS), were included in the present analysis (Table S8). Data from 30 neonates were reported for the first time in the present work. The additional 13 subjects who screened positive were recently reported by Chien et al. (2020) (12 subjects) and by Scott et al. (2020) (one subject), describing the pilot MPS IVA NBS studies in Taiwan and in Washington, respectively. The Taiwan study evaluated 73,743 neonates, detecting six subjects, with deficient GALNS enzyme activity and with biallelic *GALNS* gene variants, together with six other newborns who, harboring only one mutated *GALNS* allele were considered as carriers. The American study evaluated 106,106 DBS samples for MPS IVA, initially detecting eight samples with an enzymatic activity below 10% of the daily mean enzyme activity. Of these, only one subject was classified as a carrier of a known pathogenic variant (Scott et al., 2020).

Only one variant was detected in 25 of the 42 screening‐positive newborns identified from Taiwan overall, classifying them as possible carriers, 19 of which are reported for the first time in the present study. Among the 30 neonates who screened positive and are reported in this study for the first time, 11 had two mutated alleles.

In all screening‐positive neonates so far identified, 20 different *GALNS* variants were found, four of which reported as new in this study. As expected, for the newly identified variants, very little evidence related to their pathogenic significance was available. The analysis of genotypes from screening‐positive subjects evidenced the presence of common alleles reaching, for this group of individuals, a high incidence. The most common allele reported overall was c.857C>T [p.(Thr286Met)], detected in 33.3% of alleles identified in the Taiwanese screenings, thus likely representing a pseudo‐deficiency allele, though more evidence is needed to confirm this hypothesis. The second was c.953T>G [p.(Met318Arg)], identified in 16.7% of all characterized alleles; interestingly, this variant was reported in gnomAD only in the East‐Asian population. However, despite the high frequency in this Taiwanese NBS study, the evidence collected from the literature, as well as unpublished data from a homozygous patient affected by the severe form of the disease, support the pathogenic ACMG classification for this variant.

Data so far available from NBS highlights the need for additional molecular, biochemical, and clinical investigations, also in patients' relatives. These additional investigations are needed to evaluate the pathogenicity of the variants, to exclude possible low‐frequency polymorphisms or pseudo‐deficiency alleles, and to estimate the chances of developing the disease and its severity.

## 4 OPEN ISSUES

The description of a relatively high number of subjects with only one identified allele (127; 10.7%) or no identified alleles (12; 1.0%) highlights the fact that some variants are missed by the most common sequencing methods currently used. These genetic alterations could be large deletions, gross rearrangements, or deep intronic variants that will only come to light with second‐level molecular analyses. In addition, homozygous variants should be carefully studied. In cases where trio analyses are not available, an apparently homozygous status might be masking large deletions or, in rare cases, conditions of uniparental disomy, as well as allelic drop‐out (Caciotti et al., 2015; Catarzi et al., 2012). In any case, the set‐up of a detailed diagnostic flowchart is strongly recommended, to ensure an accurate approach in the choice of the correct molecular test for each specific suspected genetic alteration.

As most of the MPS IVA patients so far described have the classical form of the disease, and given that most of the variants are described in only one homozygous subject, the genotype‐phenotype correlation of MPS IVA is apparently straightforward. In addition, conflicting findings in patients with nonclassical forms of the disease are very rare. However, it is possible that variants associated with nonclassical phenotype may be underrepresented in this update, given that these patients may be undiagnosed. As also highlighted in the previous mutation update (Morrone, Caciotti, et al., 2014), the main clinical features on which the clinical phenotype is based remain to be height and growth. Once these factors plateau, other parameters, such as urinary‐specific GAGs (KS and C6S), range of mobility, and respiratory and cardiac manifestations, should be taken into consideration to widen the analysis and allow for an accurate clinical diagnosis and prognosis.

Finally, all variants should be annotated according to the most recent HGVS recommendations, thus allowing a consistent and unambiguous description with minimal risk of misreporting. To this aim, several in silico tools are available that may support checking and validating variants annotation (i.e., Mutalyzer—Name Checker, Variant Validator).

## 5 FUTURE PROSPECTS

The results of the first two NBS pilot programs recently reported (Chien et al., 2020; Scott et al., 2020) may encourage the expansion of NBS to wider populations, thus allowing early identification of affected children and, hence, timely intervention with enzyme replacement therapy. However, a careful long‐term follow‐up of positive cases must be guaranteed, as well as a deeper analysis of potential pseudo‐deficiency alleles. When novel variants are identified, which occurs quite commonly with NBS, we recommend pursuing the in vitro analysis of variant expression, besides enzyme activity confirmation in blood cells or fibroblasts and measurement of specific biomarkers, such as KS or C6S, with sensitive methods. Moreover, to exclude pseudo‐deficiency, a close clinical evaluation of siblings and a search for the same variant(s) in the family should also be performed. All of these analyses would help to understand more precociously the potential pathogenic significance of the variant(s).

The review of the literature since 2014 and the data from the contributing laboratories shows a greater use of NGS in the diagnosis of MPS IVA, with targeted gene panels used more frequently than whole‐exome sequencing. Several diagnoses of MPS IVA were made using large panels, including hundreds or even thousands of genes addressing specific groups of disorders (i.e., inborn errors of metabolism) or clinical signs (i.e., dysmorphology and skeletal dysplasia, disorders with orodental involvement, etc.). A few cases were diagnosed by whole‐exome sequencing or by second‐ or third‐level analyses aiming to detect gross deletions/rearrangements, copy number variations, or deep intronic variants. It is clear that the choice of molecular methods applied strictly depends on the query to be solved as well as on the technology available. It is likely that the use of NGS technologies will continue to expand given the anticipated reductions in cost and will help to molecularly define a higher number of suspects. Given the heterogenicity of disease‐associated variants in the *GALNS* gene (as shown in this update), it is important to follow up any novel variants and variants of unknown significance with evaluation of biochemical enzyme activity in leukocytes and fibroblasts, and with GAG analysis.

Increased reporting of properly classified *GALNS* variants in public databases would aid the interpretation of prenatal diagnosis, NBS findings, help to reduce time to diagnosis, prevent misdiagnosis, and assist in understanding genotype‐phenotype correlation. The availability of variants adequately analyzed in open‐source databases allows medical practitioners in all countries, including those with less developed medical care systems, to access key information and make more accurate and timely diagnoses.

## CONFLICT OF INTERESTS

Moeenaldeen AlSayed has received honorarium and travel reimbursement from BioMarin Pharmaceutical Inc., Shire, and Sanofi Genzyme. Yin‐Hsiu Chien is on the advisory board of Amicus and Sanofi, has received consulting fees from Amicus and Sanofi, has conducted research for Sanofi, and has received honoraria from Biogen, BioMarin Pharmaceutical Inc., Novartis, Sanofi, and Takeda. Roberto Giugliani has been an investigator, consultant, and/or speaker for Abeona, Allevex, Amicus, BioMarin Pharmaceutical Inc., Chiesi, Denali, Idorsia, Inventiva, JCR, Lysogene, Novartis, PassageBio, PTC, RegenxBio, Sanofi‐Genzyme, Sigilon, Sobi, Takeda, and Ultragenyx. Emanuela Izzo and Akashdeep Singh are employees of BioMarin Pharmaceutical Inc. David C. Kasper is an employee of ARCHIMED Life Science GmbH and is a stockholder in ARCHIMED Life Science GmbH. Hsiang‐Yu Lin has been an investigator, consultant, and/or speaker for BioMarin Pharmaceutical Inc., Sanofi‐Genzyme, and Takeda. Shuan‐Pei Lin is on the advisory board of HOS and Shire and has received honoraria from Sanofi Genzyme. Tim Wood is on the advisory board of Amicus‐Fabry. Amelia Morrone has been an investigator, consultant, and/or speaker for BioMarin Pharmaceutical Inc., Sanofi‐Genzyme, and Takeda. The remaining authors declare that there are no conflicts of interest.

## AUTHOR CONTRIBUTIONS

Alessandra Zanetti, Rosella Tomanin, Emanuela Izzo, Akashdeep Singh designed the project and the manuscript; Alessandra Zanetti collected and analyzed the literature and patients' data; Francesca D'Avanzo analyzed data and performed variants classification; Moeenaldeen AlSayed, Ana Carolina Brusius‐Facchin, Yin‐Hsiu Chien, Roberto Giugliani, David C. Kasper, Hsiang‐Yu Lin, Shuan‐Pei Lin, Laura Pollard, Rodolfo Tonin, Tim Wood acquired patients' data; Akashdeep Singh, Emanuela Izzo, coordinated the project and the data collection; Rosella Tomanin, Amelia Morrone supervised data analysis and interpretation; all authors critically revised and approved the manuscript before submission.

## WEB RESOURCES

ClinVar database: https://www.ncbi.nlm.nih.gov/clinvar/

Ensembl. (2020). Human (GRCh38.p13) GALNS. Retrieved from: https://www.ensembl.org/Homo_sapiens/Gene/Summary?db=core;g=ENSG00000141012;r=16:88813734-88856970

## Supporting information

Supporting information.

Supporting information.

## Data Availability

Rosella Tomanin: All disease‐associated *GALNS* variants described to date have been deposited into the ClinVar database (https://www.ncbi.nlm.nih.gov/clinvar/) at the National Center for Biotechnology Information under accession numbers SCV001547566‐SCV001548006. The full data that support the findings of this study are available in the supplementary material of this article.
