# Supplementary material for: Molecular basis of mucopolysaccharidosis IVA (Morquio A syndrome): A review and classification of GALNS gene variants and reporting of 68 novel variants
Source: Hum Mutat. 2021 Aug 23;42(11):1384–98. doi: 10.1002/humu.24270 (PMC9291100; doi:10.1002/humu.24270)
Supplement: Supplementary file 1 — Supporting information. [file HUMU-42-1384-s002.docx]

**SUPPLEMENTARY FILE S1** Sequencing methods

**DNA extraction**

Genomic DNA was extracted from peripheral whole blood using the iPrep Purelink gDNA Blood kit (Invitrogen, Carlsbad, CA, USA), the QIAsymphony DSP DNA Midi Kit (Qiagen, Hilden, Germany), the QIAamp DNA Micro Kit (Qiagen), the Flexigene DNA Kit (Qiagen), or the AGF Fresh Flexigene Reagent Kit (Autogen, Holliston, MA, USA) or from peripheral leukocytes using the QIAamp DNA Micro Kit (Qiagen). Genomic DNA from dried blood spots was extracted using the EZ1 DNA Tissue Kit (Qiagen) or the chemagic 360 instrument (PerkinElmer, Inc., Waltham, MA, USA) according to manufacturer’s instructions. Quality and quantity of extracted DNA was determined using the Nanodrop 2000 instrument (Thermo Fisher Scientific Inc., Waltham, MA, USA) and the Qubit DNA HS assay kit (Thermo Fisher Scientific Inc.).

**Target amplification/enrichment**

***Method 1 (Italy, USA, MacKay - Taiwan)***

Primers were designed for all 14 exons and flanking exon-intron boundaries of GALNS using the Primer3 Input version 0.4.0 (<http://bioinfo.ut.ee/primer3-0.4.0/>) and NCBI primer BLAST softwares (<http://www.ncbi.nlm.nih.gov/tools/primer-blast/>) (National Library of Medicine, Bethesda, MD, USA), or Primer3web version 4.1.0 (<https://primer3.ut.ee/>) for primer design, Gene Tools SNPCheck V3 (<https://genetools.org/SNPCheck/snpcheck.htm>) to ensure primers were free of SNPs, and UCSC BLAT Search (<https://genome.ucsc.edu/cgi-bin/hgBlat>) to ensure primer specificity to the intended target region. Primers sequences are available upon request.

Polymerase chain reaction (PCR) was carried out in Mastercycler^®^ nexus (Applied Biosystems, Foster City, CA, USA), Veriti 96-Well Thermal Cyclers (Applied Biosystems) or GeneAmp PCR System 9700 (Applied Biosystems) with the designed primers for the GALNS gene. Amplification of genomic fragments was performed using 75–200 ng of genomic DNA, and PCR conditions for all the exons were: denaturation at 94°C for 4–5 min, 30–35 cycles at 94°C for 30–40 s, 58–65°C for 30–40 s, 72°C for 40 s to 2 min, and a final extension cycle at 72°C for 5–10 min. PCR products were visualized on a 2% agarose gel, stained with SYBR green and visualised under an ultraviolet transilluminator. Products were purified using Nucleospin Extract II kit (Macherey-Nagel, Düren, Germany) or run on the QiAxcel (Qiagen) to verify amplification then purified using ExoSAP-IT Express PCR Product Cleanup (Applied Biosystems).

***Method 2***

Libraries of target-enriched patient DNA were established using the AmpliSeq™ library with 24 reactions (Illumina, San Diego, CA, USA), or KAPA HyperPlus Library Preparation Kit (Roche, Basel, Switzerland) following manufacturer’s instructions. The target library hybridization in solution utilized the biotinylated single-strand DNA capture probes. Streptavidin magnetic beads were used to capture the probes hybridized to the targeted regions of interest. Enriched libraries were amplified, purified and sequenced on Illumina MiniSeq sequencer with MiniSeq high output reagent kit (Illumina).

**Sanger sequencing**

Bidirectional Sanger sequencing was carried out on all the purified PCR products (method 1) on the ABI 3130 automated genetic analyzer (Applied Biosystems) or the ABI 3730xl DNA Analyzer (Applied Biosystems). In Italy, sequencing data were analyzed using the sequence analysis software, Sequencing Analysis 5.2 (Applied Biosystems), and pair-wise alignment of both the reference exonic sequence and the patient's PCR amplified exonic sequences was carried out using the BioEdit (Tom Hall, Informer Technologies, Valley, Nebraska, USA) nucleotide alignment tool for the identification of the sequence variants. In the USA, sequencing data were aligned against a reference sequence and analyzed for sequence variants using two different types of sequence analysis software: Mutation Surveyor V5.1.0 (SoftGenetics LLC, State College, PA, USA) and Sequence Pilot (JSI Medical Systems, Ettenheim, Germany).

**Next-Generation Sequencing (NGS)**

***Customized or disease-specific sequencing panels***

NGS-targeted panels covering exonic regions and intronic flanking regions of between 2 and 29 genes were used. Gene panels included an MPS panel (includes GALNS and other genes such as *ARSB*, GLB1, *IDUA, IDS, SGSH, NAGLU*), a lysosomal storage disease panel (including GALNS, ARSB, *GLA,* and *TPP1* genes, among others) and other metabolic disorders (including *ACADVL CPT1A, CPT2, DDC, ETFA, ETFB, ETFDH,* and *SLC22A5* genes, among others). The customized panels were designed using Ion Ampliseq^TM^ Designer v3.4 (Thermo Fisher Scientific), AmpliSeq for Illumina Custom Panels (Illumina) to generate a pool of primers for amplification of genomic regions of interest. All exons and flanking 10–20 bp of intron-exon junctions were amplified, followed by paired-end high-throughput sequencing using Illumina MiniSeq with coverage 150X mean depth (Illumina) or Ion Torrent Personal Genome Machine with coverage 100X mean depth (Thermo Fisher Scientific).

In Brazil, alignment and variant calling were performed according to the Torrent Suite 5.0 analysis pipeline. Variant calling was performed with the Torrent VariantCaller version 5.0 (Thermo Fisher Scientific), with default germline high-stringency parameters. Variant analysis was performed using Ion Reporter^TM^ Software (Thermo Fisher Scientific). All identified variants were verified through Integrative Genomics Viewer (IGV) version 2.3.79[11](https://www.sciencedirect.com/science/article/pii/S1525157820300179#bib11) for coverage and chromosomal position, and annotated using the Human Genome Variation Society (HGVS) [version 15.11[12](https://www.sciencedirect.com/science/article/pii/S1525157820300179#bib12)] nomenclature through Ensembl Variant Effect Predictor (<http://www.ensembl.org/info/docs/tools/vep/index.html>). In a few specific biochemically diagnosed cases, a manual analysis of the gene of interest was performed through IGV, searching for low covered variants or for homozygous deletions.

In Italy and ARCHIMED laboratories, the variant analysis was done via mapping the text files of sequence read to the reference genome UCSC hg19 using the BaseSpace Sequence Hub by DNA Amplicon (v2.1.0) (Illumina) with default parameters. Following alignment, Genome Analysis Tool Kit (GATK) software library was used to identify single nucleotide polymorphism and insertion-deletion SNPs and indels of NGS data. Then, applying public databases, including 1000 Genome Project (<http://www.1000genomes.org>) and GnomAD (http://gnomad.broadinstitute.org) variants with frequency >5% and synonymous substitutions were filtered out.

**Detection of large deletions/duplications and gross rearrangements**

Large deletions/duplications and gross rearrangements were detected by array Comparative Genomics Hybridization (aCGH) analysis.

***Method 1 (USA)***

The isolated DNA was analyzed using the Agilent 4x180K custom microarray system. This platform consists of 180,000 markers (comprised of both non-polymorphic copy number and single nucleotide polymorphism (SNP) probes).  The oligonucleotide probes are distributed across the genome with increased coverage at all the coding regions of 725 genes plus regions including common  microdeletion or microduplication syndromes. Agilent CytoGenomics edition 2.8.5.11 software  has been utilized for data analysis.. All copy number changes were determined using the human genome build 19 (hg19/NCBI build 37).

***Method 2 (Saudi Arabia)***

aCGH analysis was performed as previously reported (Kaya, 2008)

**SUPPLEMENTARY FILE S2** List of supplementary tables

**Table S1.** Specification of ACMG/AMP criteria applied to *GALNS* variants classification and/or application of the recommendations of the Sequence Variant Interpretation Working Group (SVI WG)

Abbreviations: ACMG, American College of Medical Genetics and Genomics; AMP, Association for Molecular Pathology; BA, benign stand-alone; BP, benign supporting; BS, benign strong; FGE, formylglycine-generating enzyme; LOF, loss-of-function; PM, pathogenic moderate; PP, pathogenic supporting; PS, pathogenic strong; pts, patients; PVS, pathogenic very strong; SOP, standard operating procedure; REVEL, Rare Exome Variant Ensemble Learner; WB, Western blot.

**Table S2.** List of unique *GALNS* variants

Abbreviations: UTR, untranslated region; VUS, variant of uncertain significance.

**Table S3.** Misreported variants

Abbreviations: HGVS, Human Genome Variation Society.

**Table S4.** List of reported *GALNS* genotypes from patients with Morquio A since the previous 2014 update

Abbreviations: F, female; M, male; NBS, newborn screening.

**Table S5.** List of *GALNS* genotypes provided by the collaborating laboratories

Abbreviations: F, female; M, male; NBS, newborn screening.

**Table S6.** Variants in homozygosis associated with a clinical phenotype

**Table S7.** Results of in silico analysis of novel *GALNS* variants

Abbreviations: ADA, adaptive boosting; CADD, Combined Annotation Dependent Depletion; dbscSNV, database for predicting the splicing impact of a single-nucleotide variant; dbSNP, single nucleotide polymorphism database; ExAC_all, all the data of Exome Aggregation Consortium; FATHMM, Functional Analysis through Hidden Markov Model; GERP, Genomic Evolutionary Rate Profiling; HDIV, Human Mendelian disease variants vs. divergence; PROVEAN, Protein Variation Effect Analyzer; REVEL, Rare Exome Variant Ensemble Learner; RF, random forest; RS, rejected substitution; SIFT, Sorting Intolerant From Tolerant.

**Table S8.** Genotypes of neonates positive to newborn screening

Abbreviations: F, female; M, male.

**SUPPLEMENTARY FILE S3** References for supplementary tables

Abou Tayoun, A. N., Pesaran, T., DiStefano, M. T., Oza, A., Rehm, H. L., Biesecker, L. G., Harrison, S. M., & ClinGen Sequence Variant Interpretation Working Group (ClinGen SVI). (2018). Recommendations for interpreting the loss of function PVS1 ACMG/AMP variant criterion. *Human Mutation*, *39*, 1517–1524. <https://doi.org/10.1002/humu.23626>

Aldeeri, A. A., Alazami, A. M., Hijazi, H., Alzahrani, F., & Alkuraya, F. S. (2014). Excessively redundant umbilical skin as a potential early clinical feature of Morquio syndrome and FKBP14-related Ehlers-Danlos syndrome. *Clinical Genetics*, *86*, 469–472. <https://doi.org/10.1111/cge.12414>

Alshuaibi, W., Hale, S., Petroni, D., Skoda-Smith, S., Goldberg, M. J., & Sun, A. (2016). Immunomodulation in a patient with Morquio syndrome type A treated with enzyme replacement therapy. *Molecular Genetics and Metabolism*, *117*, S18. <https://doi.org/10.1016/j.ymgme.2015.12.171>

Bidchol, A. M., Dalal, A., Shah, H., S, S., Nampoothiri, S., Kabra, M., Gupta, N., Danda, S., Gowrishankar, K., Phadke, S. R., Kapoor, S., Kamate, M., Verma, I. C., Puri, R. D., Sankar, V. H., Devi, A. R. R., Patil, S. J., Ranganath, P., Jain, S. J. M. N., Agarwal, M., Singh, A., Mishra, P., Tamhankar, P. M., Gopinath, P. M., Nagarajaram, H. A., Satyamoorthy, K., & Girisha, K. M. (2014). GALNS mutations in Indian patients with mucopolysaccharidosis IVA. *American Journal of Medical Genetics, Part A*, *164A*, 2793–2801. <https://doi.org/10.1002/ajmg.a.36735>

Biesecker, L. G., Harrison, S. M., & ClinGen Sequence Variant Interpretation Working Group. (2018). The ACMG/AMP reputable source criteria for the interpretation of sequence variants. *Genetics in Medicine*, *20*, 1687–1688. <https://doi.org/10.1038/gim.2018.42>

Bochernitsan, A. N., Brusius-Facchin, A. C., Couto, R. R., Kubaski, F., dos Santos Lopes, S. S., Gondim, C. E., de Medeiros, P. F. V., de Souza, C. F. M., Giugliani, R., & Leistner-Segal, S. (2018). Spectrum of GALNS mutations and haplotype study in Brazilian patients with mucopolysaccharidosis type IVA. *Meta Gene*, *16*, 77–84. <https://doi.org/10.1016/j.mgene.2018.01.008>

Bunge, S., Kleijer, W. J., Tylki-Szymanska, A., Steglich, C., Beck, M., Tomatsu, S., Fukuda, S., Poorthuis, B. J., Czartoryska, B., Orii, T., & Gal, A. (1997). Identification of 31 novel mutations in the N-acetylgalactosamine-6-sulfatase gene reveals excessive allelic heterogeneity among patients with Morquio A syndrome. *Human Mutation*, *10*, 223–232. [https://doi.org/10.1002/(SICI)1098-1004(1997)10:3<223::AID-HUMU8>3.0.CO;2-J](https://doi.org/10.1002/(SICI)1098-1004(1997)10:3%3c223::AID-HUMU8%3e3.0.CO;2-J)

Caciotti, A., Tonin, R., Mort, M., Cooper, D. N., Gasperini, S., Rigoldi, M., Parini, R., Deodato, F., Taurisano, R., Sibilio, M., Parenti, G., Guerrini, R., & Morrone, A. (2018). Mis-splicing of the GALNS gene resulting from deep intronic mutations as a cause of Morquio A disease. *BMC Medical Genetics*, *19*, 183. <https://doi.org/10.1186/s12881-018-0694-6>

Caciotti, A., Tonin, R., Rigoldi, M., Ferri, L., Catarzi, S., Cavicchi, C., Procopio, E., Donati, M. A., Ficcadenti, A., Fiumara, A., Barone, R., Garavelli, L., Rocco, M. D., Filocamo, M., Antuzzi, D., Scarpa, M., Mooney, S. D., Li, B., Skouma, A., Bianca, S., Concolino, D., Casalone, R., Monti, E., Pantaleo, M., Giglio, S., Guerrini, R., Parini, R., & Morrone, A. (2015). Optimizing the molecular diagnosis of GALNS: Novel methods to define and characterize Morquio-A syndrome-associated mutations. *Human Mutation*, *36*, 357–368. <https://doi.org/10.1002/humu.22751>

Carraresi, L., Parini, R., Filoni, C., Caciotti, A., Sersale, G., Tomatsu, S., Orlando, C., Zammarchi, E., Guerrini, R., Donati, M. A., & Morrone, A. (2008). GALNS gene expression profiling in Morquio A patients' fibroblasts. *Clinica Chimica Acta*, *397*, 72–76. <https://doi.org/10.1016/j.cca.2008.07.021>

Catarzi, S., Giunti, L., Papadia, F., Gabrielli, O., Guerrini, R., Donati, M. A., Genuardi, M., & Morrone, A. (2012). Morquio A syndrome due to maternal uniparental isodisomy of the telomeric end of chromosome 16. *Molecular Genetics and Metabolism*, *105*, 438–442. <https://doi.org/10.1016/j.ymgme.2011.11.196>

Chen, Q., Chen, Y., Liu, X., & Wei, H. (2017). [Analysis of clinical features and GALNS gene mutation in a patient with mucopolysaccharidosis type IV A]. *Chinese Journal of Medical Genetics/Zhonghua Yi Xue Yi Chuan Xue Za Zhi*, *34*, 232–235. <https://doi.org/10.3760/cma.j.issn.1003-9406.2017.02.018>

Chien, Y.-H., Lee, N.-C., Chen, P.-W., Yeh, H.-Y., Gelb, M. H., Chiu, P.-C., Chu, S.-Y., Lee, C.-H., Lee, A.-R., & Hwu, W.-L. (2020). Newborn screening for Morquio disease and other lysosomal storage diseases: Results from the 8-plex assay for 70,000 newborns. *Orphanet Journal of Rare Diseases*, *15*, 38. <https://doi.org/10.1186/s13023-020-1322-z>

Chin, S. J., Saville, J. T., McDermott, B. K., Zankl, A., Fletcher, J. M., & Fuller, M. (2020). Chondroitin sulfate disaccharide is a specific and sensitive biomarker for mucopolysaccharidosis type IVA. *JIMD Reports*, *55*, 68–74. <https://doi.org/10.1002/jmd2.12132>

Chistiakov, D. A., Savost'anov, K. V., Kuzenkova, L. M., Gevorkyan, A. K., Pushkov, A. A., Nikitin, A. G., Pakhomov, A. V., Vashakmadze, N. D., Zhurkova, N. V., Podkletnova, T. V., Mayansky, N. A., Namazova-Baranova, L. S., & Baranov, A. A. (2014). Molecular characteristics of patients with glycosaminoglycan storage disorders in Russia. *Clinica Chimica Acta*, *436*, 112–120. <https://doi.org/10.1016/j.cca.2014.05.010>

Chkioua, L., Khedhiri, S., Hafsi, H., Grissa, O., Ben Turkia, H., Miled, A., Laradi, S., Froissart, R., & Alif, N. (2016). Molecular analysis in a GALNS study cohort of 15 Tunisian patients: Description of a novel mutation. *Diagnostic Pathology*, *11*, 51. <https://doi.org/10.1186/s13000-016-0498-y>

Clinical Genome Resource. (2019). ClinGen general sequence variant curation process - standard operating procedure. <https://clinicalgenome.org/site/assets/files/3677/clingen_variant-curation_sopv1.pdf>

Cole, D. E., Fukuda, S., Gordon, B. A., Rip, J. W., LeCouteur, A. N., Rupar, C. A., Tomatsu, S., Ogawa, T., Sukegawa, K., & Orii, T. (1996). Heteroallelic missense mutations of the galactosamine-6-sulfate sulfatase (GALNS) gene in a mild form of Morquio disease (MPS IVA). *American Journal of Medical Genetics*, *63*, 558–565. [https://doi.org/10.1002/(SICI)1096-8628(19960628)63:4<558::AID-AJMG9>3.0.CO;2-K](https://doi.org/10.1002/(SICI)1096-8628(19960628)63:4%3c558::AID-AJMG9%3e3.0.CO;2-K)

Cozma, C., Eichler, S., Wittmann, G., Flores Bonet, A., Kramp, G. J., Giese, A.-K., & Rolfs, A. (2015). Diagnosis of Morquio syndrome in dried blood spots based on a New MRM-MS assay. *PLoS ONE*, *10*, e0131228. <https://doi.org/10.1371/journal.pone.0131228>

Dieter, T., Matte, U. d. S., Schwartz, I. V., Tomatsu, S., & Giugliani, R. (2007). Common N-acetylgalactosamine-6-sulfate sulfatase (GALNS) exon mutations in Brazilian patients with mucopolysaccharidosis IVA (MPS IVA). *Genetics and Molecular Biology*, *30*, 524–528. <http://dx.doi.org/10.1590/S1415-47572007000400004>

Dũng, V. C., Tomatsu, S., Montaño, A. M., Gottesman, G., Bober, M. B., Mackenzie, W., Maeda, M., Mitchell, G. A., Suzuki, Y., & Orii, T. (2013). Mucopolysaccharidosis IVA: Correlation between genotype, phenotype and keratan sulfate levels. *Molecular Genetics and Metabolism*, *110*, 129–138. <https://doi.org/10.1016/j.ymgme.2013.06.008>

Fateen, E. M., El Mawgoud, H. A., Eissa, N. R., Ibrahim, M. M., Aglan, M. S., & Essawi, M. L. (2017). Four novel mutations in the N-acetylgalactosamine-6-sulfate sulfatase gene among Egyptian patients with Morquio A disease. *Gene*, *600*, 48–54. <https://doi.org/10.1016/j.gene.2016.11.002>

Ficicioglu, C., Matalon, D. R., Luongo, N., Menello, C., Kornafel, T., & Degnan, A. J. (2020). Diagnostic journey and impact of enzyme replacement therapy for mucopolysaccharidosis IVA: A sibling control study. *Orphanet Journal of Rare Diseases*, *15*, 336–336. <https://doi.org/10.1186/s13023-020-01618-y>

Fukuda, S., Tomatsu, S., Cooper, A., Wraith, J. E., Kato, Z., Yamada, N., Isogai, K., Sukegawa, K., Kondo, N., & Orii, T. (1996). Mucopolysaccharidosis IVA (Morquio A): Three novel small deletions in the N-acetylgalactosamine-6-sulfate sulfatase gene. *Human Mutation*, *8*, 187–190. [https://doi.org/10.1002/(SICI)1098-1004(1996)8:2<187::AID-HUMU14>3.0.CO;2-Z](https://doi.org/10.1002/(SICI)1098-1004(1996)8:2%3c187::AID-HUMU14%3e3.0.CO;2-Z)

Fukuda, S., Tomatsu, S., Masue, M., Sukegawa, K., Iwata, H., Ogawa, T., Nakashima, Y., Hori, T., Yamagishi, A., & Hanyu, Y. (1992). Mucopolysaccharidosis type IVA. N-acetylgalactosamine-6-sulfate sulfatase exonic point mutations in classical Morquio and mild cases. *The Journal of Clinical Investigation*, *90*, 1049–1053. <https://doi.org/10.1172/JCI115919>

Fukuda, S., Tomatsu, S., Masuno, M., Ogawa, T., Yamagishi, A., Rezvi, G. M., Sukegawa, K., Shimozawa, N., Suzuki, Y., Kondo, N., Imaizumi, K., Kuroki, Y., Okabe, T., & Orii, T. (1996). Mucopolysaccharidosis IVA: Submicroscopic deletion of 16q24.3 and a novel R386C mutation of N-acetylgalactosamine-6-sulfate sulfatase gene in a classical Morquio disease. *Human Mutation*, *7*, 123–134. [https://doi.org/10.1002/(SICI)1098-1004(1996)7:2<123::AID-HUMU6>3.0.CO;2-D](https://doi.org/10.1002/(SICI)1098-1004(1996)7:2%3c123::AID-HUMU6%3e3.0.CO;2-D)

Fukuda, S., Yamada, N., Tomatsu, S., Sukegawa, K., Montaño, A. M., Hopwood, J. J., Muller, V., Orii, T., & Kondo, N. (1997). Mucopolysaccharidosis IVA: A novel splice acceptor site mutation in intron 4 of the N-acetylgalactosamine-6-sulfate sulfatase gene in an Afghanistan girl with classical Morquio disease. *The Japanese Journal of Human Genetics*, *42*, 317–322. <https://doi.org/10.1007/BF02766953>

Ghosh, R., Harrison, S. M., Rehm, H. L., Plon, S. E., & Biesecker, L. G. (2018). Updated recommendation for the benign stand-alone ACMG/AMP criterion. *Human Mutation*, *39*, 1525–1530. <https://doi.org/10.1002/humu.23642>

Guo, Y.-b., Ai, Y., Zhao, Y., Tang, J., Jiang, W.-y., Du, M.-l., Ma, H.-m., & Zhong, Y.-f. (2012). [Rapid prenatal genetic diagnosis of a fetus with a high risk for Morquio A syndrome]. *Chinese Journal of Medical Genetics/Zhonghua Yi Xue Yi Chuan Xue Za Zhi*, *29*, 126–130. <https://doi.org/10.3760/cma.j.issn.1003-9406.2012.02.002>

He, D., Huang, Y., Ou, Z., Sheng, H., Li, S., Zhao, X., Li, R., Zheng, J., & Liu, L. (2013). Molecular genetic assay of mucopolysaccharidosis IVA in South China. *Gene*, *532*, 46–52. <https://doi.org/10.1016/j.gene.2013.08.097>

Hori, T., Tomatsu, S., Nakashima, Y., Uchiyama, A., Fukuda, S., Sukegawa, K., Shimozawa, N., Suzuki, Y., Kondo, N., & Horiuchi, T. (1995). Mucopolysaccharidosis type IVA: Common double deletion in the N-acetylgalactosamine-6-sulfatase gene (GALNS). *Genomics*, *26*, 535–542. <https://doi.org/10.1016/0888-7543(95)80172-i>

Huang, Z., Sun, Y., Fan, Y., Wang, L., Liu, H., Gong, Z., Wang, J., Yan, H., Wang, Y., Hu, G., Wang, R., Ye, J., Han, L., Qiu, W., Zhang, H., Liang, L., Yang, Y., Dauber, A., Yu, Y., & Gu, X. F. (2018). Genetic evaluation of 114 Chinese short stature children in the next generation era: A single center study. *Cellular Physiology and Biochemistry*, *49*, 295–305. <https://doi.org/10.1159/000492879>

Ioannidis, N. M., Rothstein, J. H., Pejaver, V., Middha, S., McDonnell, S. K., Baheti, S., Musolf, A., Li, Q., Holzinger, E., Karyadi, D., Cannon-Albright, L. A., Teerlink, C. C., Stanford, J. L., Isaacs, W. B., Xu, J., Cooney, K. A., Lange, E. M., Schleutker, J., Carpten, J. D., Powell, I. J., Cussenot, O., Cancel-Tassin, G., Giles, G. G., MacInnis, R. J., Maier, C., Hsieh, C.-L., Wiklund, F., Catalona, W. J., Foulkes, W. D., Mandal, D., Eeles, R. A., Kote-Jarai, Z., Bustamante, C. D., Schaid, D. J., Hastie, T., Ostrander, E. A., Bailey-Wilson, J. E., Radivojac, P., Thibodeau, S. N., Whittemore, A. S., & Sieh, W. (2016). REVEL: An ensemble method for predicting the pathogenicity of rare missense variants. *American Journal of Human Genetics*, *99*, 877–885. <https://doi.org/10.1016/j.ajhg.2016.08.016>

Jezela-Stanek, A., Różdżyńska-Świątkowska, A., Kulpanovich, A., Ciara, E., Marucha, J., & Tylki-Szymańska, A. (2019). Novel data on growth phenotype and causative genotypes in 29 patients with Morquio (Morquio-Brailsford) syndrome from Central-Eastern Europe. *Journal of Applied Genetics*, *60*, 163–174. <https://doi.org/10.1007/s13353-019-00491-1>

Kato, Z., Fukuda, S., Tomatsu, S., Vega, H., Yasunaga, T., Yamagishi, A., Yamada, N., Valencia, A., Barrera, L. A., Sukegawa, K., Orii, T., & Kondo, N. (1997). A novel common missense mutation G301C in the N-acetylgalactosamine-6-sulfate sulfatase gene in mucopolysaccharidosis IVA. *Human Genetics*, *101*, 97–101. <https://doi.org/10.1007/s004390050594>

Kaya, N., Al-Owain, M., Albakheet, A., Colak, D., Al-Odaib, A., Imtiaz, F., Coskun, S., Al-Sayed, M., Al-Hassnan, Z., Al-Zaidan, H., Meyer B., & Ozand, P. (2008). Array comparative genomic hybridization (aCGH) reveals the largest novel deletion in PCCA found in a Saudi family with propionic acidemia. *Eur J Med Genet*, *51*, 558−565. https://doi.org/10.1016/j.ejmg.2008.08.001

Khedhiri, S., Chkioua, L., Bouzidi, H., Dandana, A., Ferchichi, S., Ben Turkia, H., Miled, A., & Laradi, S. (2012). Mucopolysaccharidosis IVA within Tunisian patients: Confirmation of the two novel GALNS gene mutations. *Pathologie Biologie*, *60*, 190–192. <https://doi.org/10.1016/j.patbio.2011.03.001>

Khedhiri, S., Chkioua, L., Elcioglu, N., Laradi, S., & Miled, A. (2014). Mutations and polymorphisms in N-acetylgalactosamine-6-sulfate sulfatase gene in Turkish Morquio A patients. *Pathologie Biologie*, *62*, 38–40. <https://doi.org/10.1016/j.patbio.2013.10.001>

Khedhiri, S., Chkioua, L., Ferchichi, S., Miled, A., & Laradi, S. (2011). Morquio A disease: Clinical and molecular study of Tunisian patients [in French]. *Annales de Biologie Clinique*, *69*, 425–429. <https://doi.org/10.1684/abc.2011.0593>

Khoshaeen, A., Mahdavi, M. R., Najafi, M., Jalali, H., & Mahdavi, M. (2019). An ultra-rare mutation (C.181C>T) in GALNS gene associated with Morquio syndrome: A case report. *Gene Reports*, *17*, 100488. <https://doi.org/10.1016/j.genrep.2019.100488>

Kubaski, F., Brusius-Facchin, A. C., Palhares, H. M. C., Balarin, M. A. S., Viapiana-Camelier, M., Guidobono, R., Burin, M. G., Giugliani, R., & Leistner-Segal, S. (2013). Identification of a novel missense mutation in Brazilian patient with a severe form of mucopolysaccharidosis type IVA. *Gene*, *517*, 112–115. <https://doi.org/10.1016/j.gene.2012.12.100>

Lachman, R. S., Burton, B. K., Clarke, L. A., Hoffinger, S., Ikegawa, S., Jin, D.-K., Kano, H., Kim, O.-H., Lampe, C., Mendelsohn, N. J., Shediac, R., Tanpaiboon, P., & White, K. K. (2014). Mucopolysaccharidosis IVA (Morquio A syndrome) and VI (Maroteaux-Lamy syndrome): Under-recognized and challenging to diagnose. *Skeletal Radiology*, *43*, 359–369. <https://doi.org/10.1007/s00256-013-1797-y>

Laradi, S., Tukel, T., Khediri, S., Shabbeer, J., Erazo, M., Chkioua, L., Chaabouni, M., Ferchichi, S., Miled, A., & Desnick, R. J. (2006). Mucopolysaccharidosis type IV: N-acetylgalactosamine-6-sulfatase mutations in Tunisian patients. *Molecular Genetics and Metabolism*, *87*, 213–218. <https://doi.org/10.1016/j.ymgme.2005.11.001>

Lee, N. H., Cho, S. Y., Maeng, S. H., Jeon, T. Y., Sohn, Y. B., Kim, S. J., Park, H.-D., & Jin, D. K. (2012). Clinical, radiologic, and genetic features of Korean patients with mucopolysaccharidosis IVA. *Korean Journal of Pediatrics*, *55*, 430–437. <https://doi.org/10.3345/kjp.2012.55.11.430>

Leong, H. Y., Abdul Azize, N. A., Chew, H. B., Keng, W. T., Thong, M. K., Mohd Khalid, M. K. N., Hung, L. C., Mohamed Zainudin, N., Ramlee, A., Md Haniffa, M. A., Yakob, Y., & Ngu, L. H. (2019). Clinical, biochemical and genetic profiles of patients with mucopolysaccharidosis type IVA (Morquio A syndrome) in Malaysia: The first national natural history cohort study. *Orphanet Journal of Rare Diseases*, *14*, 143. <https://doi.org/10.1186/s13023-019-1105-6>

Li, R., Baskfield, A., Beers, J., Zou, J., Liu, C., Alméciga-Díaz, C. J., & Zheng, W. (2019). Generation of an induced pluripotent stem cell line (TRNDi005-A) from a mucopolysaccharidosis type IVA (MPS IVA) patient carrying compound heterozygous p.R61W and p.WT405del mutations in the GALNS gene. *Stem Cell Research*, *36*, 101408. <https://doi.org/10.1016/j.scr.2019.101408>

Maddirevula, S., Alsahli, S., Alhabeeb, L., Patel, N., Alzahrani, F., Shamseldin, H. E., Anazi, S., Ewida, N., Alsaif, H. S., Mohamed, J. Y., Alazami, A. M., Ibrahim, N., Abdulwahab, F., Hashem, M., Abouelhoda, M., Monies, D., Al Tassan, N., Alshammari, M., Alsagheir, A., Seidahmed, M. Z., Sogati, S., Aglan, M. S., Hamad, M. H., Salih, M. A., Hamed, A. A., Alhashmi, N., Nabil, A., Alfadli, F., Abdel-Salam, G. M. H., Alkuraya, H., Peitee, W. O., Keng, W. T., Qasem, A., Mushiba, A. M., Zaki, M. S., Fassad, M. R., Alfadhel, M., Alexander, S., Sabr, Y., Temtamy, S., Ekbote, A. V., Ismail, S., Hosny, G. A., Otaify, G. A., Amr, K., Al Tala, S., Khan, A. O., Rizk, T., Alaqeel, A., Alsiddiky, A., Singh, A., Kapoor, S., Alhashem, A., Faqeih, E., Shaheen, R., & Alkuraya, F. S. (2018). Expanding the phenome and variome of skeletal dysplasia. *Genetics in Medicine*, *20*, 1609–1616. <https://doi.org/10.1038/gim.2018.50>

Moisan, L., Iannuzzi, D., Maranda, B., Campeau, P. M., & Mitchell, J. J. (2020). Clinical characteristics of patients from Quebec, Canada, with Morquio A syndrome: A longitudinal observational study. *Orphanet Journal of Rare Diseases*, *15*, 270. <https://doi.org/10.1186/s13023-020-01545-y>

Montaño, A. M., Kaitila, I., Sukegawa, K., Tomatsu, S., Kato, Z., Nakamura, H., Fukuda, S., Orii, T., & Kondo, N. (2003). Mucopolysaccharidosis IVA: Characterization of a common mutation found in Finnish patients with attenuated phenotype. *Human Genetics*, *113*, 162–169. <https://doi.org/10.1007/s00439-003-0959-8>

Montaño, A. M., Sukegawa, K., Kato, Z., Carrozzo, R., Di Natale, P., Christensen, E., Orii, K. O., Orii, T., Kondo, N., & Tomatsu, S. (2007). Effect of 'attenuated' mutations in mucopolysaccharidosis IVA on molecular phenotypes of N-acetylgalactosamine-6-sulfate sulfatase. *Journal of Inherited Metabolic Disease*, *30*, 758–767. <https://doi.org/10.1007/s10545-007-0702-z>

Moreno Giraldo, L. J., Escudero Rodríguez, Á. M., Sánchez Gómez, A., & Satizabal Soto, J. M. (2018). Clinical and molecular characteristics of Colombian patients with mucopolysaccharidosis IVA, and description of a new GALNS gene mutation. *Molecular Genetics and Metabolism Reports*, *16*, 53–56. <https://doi.org/10.1016/j.ymgmr.2018.06.008>

Morrone, A., Caciotti, A., Atwood, R., Davidson, K., Du, C., Francis-Lyon, P., Harmatz, P., Mealiffe, M., Mooney, S., Oron, T. R., Ryles, A., Zawadzki, K. A., & Miller, N. (2014). Morquio A syndrome-associated mutations: A review of alterations in the GALNS gene and a new locus-specific database. *Human Mutation*, *35*, 1271–1279. <https://doi.org/10.1002/humu.22635>

Morrone, A., Tylee, K. L., Al-Sayed, M., Brusius-Facchin, A. C., Caciotti, A., Church, H. J., Coll, M. J., Davidson, K., Fietz, M. J., Gort, L., Hegde, M., Kubaski, F., Lacerda, L., Laranjeira, F., Leistner-Segal, S., Mooney, S., Pajares, S., Pollard, L., Ribeiro, I., Wang, R. Y., & Miller, N. (2014). Molecular testing of 163 patients with Morquio A (mucopolysaccharidosis IVA) identifies 39 novel GALNS mutations. *Molecular Genetics and Metabolism*, *112*, 160–170. <https://doi.org/10.1016/j.ymgme.2014.03.004>

Nakamura-Utsunomiya, A., Nakamae, T., Kagawa, R., Karakawa, S., Sakata, S., Sakura, F., Tani, C., Matsubara, Y., Ishino, T., Tajima, G., & Okada, S. (2020). A case report of a Japanese boy with Morquio A syndrome: Effects of enzyme replacement therapy initiated at the age of 24 months. *International Journal of Molecular Sciences*, *21*, 989. <https://doi.org/10.3390/ijms21030989>

Ogawa, T., Tomatsu, S., Fukuda, S., Yamagishi, A., Rezvi, G. M. M., Sukegawa, K., Kondo, N., Suzuki, Y., Shimozawa, N., & Orü, T. (1995). Mucopolysaccharidosis IVA: Screening and identification of mutations of the N-acetylgalactosamine-6-sulfate sulfatase gene. *Human Molecular Genetics*, *4*, 341–349. <https://doi.org/10.1093/hmg/4.3.341>

Pajares, S., Alcalde, C., Couce, M. L., Del Toro, M., González-Meneses, A., Guillén, E., Pineda, M., Pintos, G., Gort, L., & Coll, M. J. (2012). Molecular analysis of mucopolysaccharidosis IVA (Morquio A) in Spain. *Molecular Genetics and Metabolism*, *106*, 196–201. <https://doi.org/10.1016/j.ymgme.2012.03.006>

Park, H.-D., Ko, A.-R., Ki, C.-S., Lee, S.-Y., Kim, J.-W., Cho, S. Y., Kim, S. H., Park, S. W., Sohn, Y. B., & Jin, D.-K. (2013). Five novel mutations of GALNS in Korean patients with mucopolysaccharidosis IVA. *American Journal of Medical Genetics, Part A*, *161*, 509–517. <https://doi.org/10.1002/ajmg.a.35298>

Peretz, R. H., Flora, C. H., & Adams, D. J. (2020). Natural history of the oldest known females with mucopolysaccharidosis type IVA (Morquio A syndrome). *American Journal of Medical Genetics, Part A*, *182*, 1491–1495. <https://doi.org/10.1002/ajmg.a.61566>

Pintos-Morell, G., Blasco-Alonso, J., Couce, M. L., Gutiérrez-Solana, L. G., Guillén-Navarro, E., O'Callaghan, M., & del Toro, M. (2018). Elosulfase alfa for mucopolysaccharidosis type IVA: Real-world experience in 7 patients from the Spanish Morquio-A early access program. *Molecular Genetics and Metabolism Reports*, *15*, 116–120. <https://doi.org/10.1016/j.ymgmr.2018.03.009>

Pollard, L. M., Jones, J. R., & Wood, T. C. (2013). Molecular characterization of 355 mucopolysaccharidosis patients reveals 104 novel mutations. *Journal of Inherited Metabolic Disease*, *36*, 179–187. <https://doi.org/10.1007/s10545-012-9533-7>

Prasad, M. K., Geoffroy, V., Vicaire, S., Jost, B., Dumas, M., Le Gras, S., Switala, M., Gasse, B., Laugel-Haushalter, V., Paschaki, M., Leheup, B., Droz, D., Dalstein, A., Loing, A., Grollemund, B., Muller-Bolla, M., Lopez-Cazaux, S., Minoux, M., Jung, S., Obry, F., Vogt, V., Davideau, J.-L., Davit-Beal, T., Kaiser, A.-S., Moog, U., Richard, B., Morrier, J.-J., Duprez, J.-P., Odent, S., Bailleul-Forestier, I., Rousset, M. M., Merametdijan, L., Toutain, A., Joseph, C., Giuliano, F., Dahlet, J.-C., Courval, A., El Alloussi, M., Laouina, S., Soskin, S., Guffon, N., Dieux, A., Doray, B., Feierabend, S., Ginglinger, E., Fournier, B., de la Dure Molla, M., Alembik, Y., Tardieu, C., Clauss, F., Berdal, A., Stoetzel, C., Manière, M. C., Dollfus, H., & Bloch-Zupan, A. (2016). A targeted next-generation sequencing assay for the molecular diagnosis of genetic disorders with orodental involvement. *Journal of Medical Genetics*, *53*, 98–110. <https://doi.org/10.1136/jmedgenet-2015-103302>

Qubbaj, W., Al-Aqeel, A. I., Al-Hassnan, Z., Al-Duraihim, A., Awartani, K., Al-Rejjal, R., & Coskun, S. (2008). Preimplantation genetic diagnosis of Morquio disease. *Prenatal Diagnosis*, *28*, 900–903. <https://doi.org/10.1002/pd.2081>

Rivera-Colón, Y., Schutsky, E. K., Kita, A. Z., & Garman, S. C. (2012). The structure of human GALNS reveals the molecular basis for mucopolysaccharidosis IV A. *Journal of Molecular Biology*, *423*, 736–751. <https://doi.org/10.1016/j.jmb.2012.08.020>

Romdhane, L., Kefi, R., Azaiez, H., Ben Halim, N., Dellagi, K., & Abdelhak, S. (2012). Founder mutations in Tunisia: Implications for diagnosis in North Africa and Middle East. *Orphanet Journal of Rare Diseases*, *7*, 52. <https://doi.org/10.1186/1750-1172-7-52>

Rush, E. T. (2016). Atypical presentation of mucopolysaccharidosis type IVA. *Molecular Genetics and Metabolism Reports*, *8*, 8–12. <https://doi.org/10.1016/j.ymgmr.2016.05.006>

Saudi Mendeliome Group. (2015). Comprehensive gene panels provide advantages over clinical exome sequencing for Mendelian diseases. *Genome Biology*, *16*, 134. <https://doi.org/10.1186/s13059-015-0693-2>

Scott, C. R., Elliott, S., Hong, X., Huang, J.-Y., Kumar, A. B., Yi, F., Pendem, N., Chennamaneni, N. K., & Gelb, M. H. (2020). Newborn screening for mucopolysaccharidoses: Results of a pilot study with 100 000 dried blood spots. *The Journal of Pediatrics*, *216*, 204–207. <https://doi.org/10.1016/j.jpeds.2019.09.036>

Seyedhassani, S. M., Hashemi-Gorji, F., Yavari, M., & Mirfakhraie, R. (2015). Novel missense mutation in the GALNS gene in an affected patient with severe form of mucopolysaccharidosis type IVA. *Clinica Chimica Acta*, *450*, 121–124. <https://doi.org/10.1016/j.cca.2015.08.006>

Smit, A. F. A., Hubley, R., & Green, P. (2019). RepeatMasker open-3.0. <http://www.repeatmasker.org/>

Sukegawa, K., Nakamura, H., Kato, Z., Tomatsu, S., Montaño, A. M., Fukao, T., Toietta, G., Tortora, P., Orii, T., & Kondo, N. (2000). Biochemical and structural analysis of missense mutations in N-acetylgalactosamine-6-sulfate sulfatase causing mucopolysaccharidosis IVA phenotypes. *Human Molecular Genetics*, *9*, 1283–1290. <https://doi.org/10.1093/hmg/9.9.1283>

Sun, A., Alshuaibi, W., Petroni, D., Skoda-Smith, S., Goldberg, M. J., & Hale, S. (2018). Immune modulation in a patient with Morquio syndrome treated with enzyme replacement therapy. *Journal of Allergy and Clinical Immunology*, *6*, 1749–1751. <https://doi.org/10.1016/j.jaip.2017.12.024>

Szklanny, K., Gubrynowicz, R., & Tylki-Szymańska, A. (2018). Voice alterations in patients with Morquio A syndrome. *Journal of Applied Genetics*, *59*, 73–80. <https://doi.org/10.1007/s13353-017-0421-6>

Tapiero-Rodriguez, S. M., Acosta Guio, J. C., Porras-Hurtado, G. L., García, N., Solano, M., Pachajoa, H., & Velasco, H. M. (2018). Determination of genotypic and clinical characteristics of Colombian patients with mucopolysaccharidosis IVA. *The Application of Clinical Genetics*, *11*, 45–57. <https://doi.org/10.2147/TACG.S141881>

Terzioglu, M., Tokatli, A., Coskun, T., & Emre, S. (2002). Molecular analysis of Turkish mucopolysaccharidosis IVA (Morquio A) patients: Identification of novel mutations in the N-acetylgalactosamine-6-sulfate sulfatase (GALNS) gene. *Human Mutation*, *20*, 477–478. <https://doi.org/10.1002/humu.9088>

Tomatsu, S., Dieter, T., Schwartz, I. V., Sarmient, P., Giugliani, R., Barrera, L. A., Guelbert, N., Kremer, R., Repetto, G. M., Gutierrez, M. A., Nishioka, T., Serrato, O. P., Montaño, A. M., Yamaguchi, S., & Noguchi, A. (2004). Identification of a common mutation in mucopolysaccharidosis IVA: Correlation among genotype, phenotype, and keratan sulfate. *Journal of Human Genetics*, *49*, 490–494. <https://doi.org/10.1007/s10038-004-0178-8>

Tomatsu, S., Filocamo, M., Orii, K. O., Sly, W. S., Gutierrez, M. A., Nishioka, T., Serrato, O. P., Di Natale, P., Montaño, A. M., Yamaguchi, S., Kondo, N., Orii, T., & Noguchi, A. (2004). Mucopolysaccharidosis IVA (Morquio A): Identification of novel common mutations in the N-acetylgalactosamine-6-sulfate sulfatase (GALNS) gene in Italian patients. *Human Mutation*, *24*, 187–188. <https://doi.org/10.1002/humu.9265>

Tomatsu, S., Fukuda, S., Cooper, A., Wraith, J. E., Ferreira, P., Di Natale, P., Tortora, P., Fujimoto, A., Kato, Z., Yamada, N., Isogai, K., Yamagishi, A., Sukegawa, K., Suzuki, Y., Shimozawa, N., Kondo, N., Sly, W. S., & Orii, T. (1997). Fourteen novel mucopolysaccharidosis IVA producing mutations in GALNS gene. *Human Mutation*, *10*, 368–375. [https://doi.org/10.1002/(SICI)1098-1004(1997)10:5<368::AID-HUMU6>3.0.CO;2-B](https://doi.org/10.1002/(SICI)1098-1004(1997)10:5%3c368::AID-HUMU6%3e3.0.CO;2-B)

Tomatsu, S., Fukuda, S., Cooper, A., Wraith, J. E., Rezvi, G. M. M., Yamagishi, A., Yamada, N., Kato, Z., Isogai, K., Sukegawa, K., Kondo, N., Suzuki, Y., Shimozawa, N., & Orii, T. (1995). Mucopolysaccharidosis type IVA: Identification of six novel mutations among non-Japanese patients. *Human Molecular Genetics*, *4*, 741–743. <https://doi.org/10.1093/hmg/4.4.741>

Tomatsu, S., Fukuda, S., Ogawa, T., Kato, Z., Isogal, K., Kondo, N., Suzuki, Y., Shimozawa, N., Sukegawa, K., & Orii, T. (1994). A novel splice site mutation intron 1 of the GALNS gene in a Japanese patient with mucopolysaccharidosis IVA. *Human Molecular Genetics*, *3*, 1427–1428. <https://doi.org/10.1093/hmg/3.8.1427>

Tomatsu, S., Fukuda, S., Yamagishi, A., Cooper, A., Wraith, J. F., Hori, T., Kato, Z., Yamada, N., Isogai, K., Sukegawa, K., Kondo, N., Suzuki, Y., Shimozawa, N., & Orii, T. (1996). Mucopolysaccharidosis IVA: Four new exonic mutations in patients with N-acetylgalactosamine-6-sulfate sulfatase deficiency. *American Journal of Human Genetics*, *58*, 950–962.

Tomatsu, S., Montaño, A. M., Nishioka, T., Gutierrez, M. A., Peña, O. M., Tranda Firescu, G. G., Lopez, P., Yamaguchi, S., Noguchi, A., & Orii, T. (2005). Mutation and polymorphism spectrum of the GALNS gene in mucopolysaccharidosis IVA (Morquio A). *Human Mutation*, *26*, 500–512. <https://doi.org/10.1002/humu.20257>

Tomatsu, S., Nishioka, T., Montaño, A. M., Gutierrez, M. A., Pena, O. S., Orii, K. O., Sly, W. S., Yamaguchi, S., Orii, T., Paschke, E., Kircher, S. G., & Noguchi, A. (2004). Mucopolysaccharidosis IVA: Identification of mutations and methylation study in GALNS gene. *Journal of Medical Genetics*, *41*, e98. <https://doi.org/10.1136/jmg.2003.018010>

Tulebayeva, A., Sharipova, M., & Boranbayeva, R. (2020). Respiratory dysfunction in children and adolescents with mucopolysaccharidosis types I, II, IVA, and VI. *Diagnostics (Basel, Switzerland)*, *10*, 63. <https://doi.org/10.3390/diagnostics10020063>

Tüysüz, B., Alkaya, D. U., Toksoy, G., Güneş, N., Yıldırım, T., Bayhan, İ. A., & Uyguner, Z. O. (2019). Mutation spectrum and pivotal features for differential diagnosis of mucopolysaccharidosis IVA patients with severe and attenuated phenotype. *Gene*, *704*, 59–67. <https://doi.org/10.1016/j.gene.2019.04.026>

Tylki-Szymańska, A., Czartoryska, B., Bunge, S., van Diggelen, O. P., Kleijer, W. J., Poorthuis, B. J., Huijmans, J. G., & Górska, D. (1998). Clinical, biochemical and molecular findings in a two-generation Morquio A family. *Clinical Genetics*, *53*, 369–374. <https://doi.org/10.1111/j.1399-0004.1998.tb02747.x>

Walsh, R., Lahrouchi, N., Tadros, R., Kyndt, F., Glinge, C., Postema, P. G., Amin, A. S., Nannenberg, E. A., Ware, J. S., Whiffin, N., Mazzarotto, F., Škorić-Milosavljević, D., Krijger, C., Arbelo, E., Babuty, D., Barajas-Martinez, H., Beckmann, B. M., Bézieau, S., Bos, J. M., Breckpot, J., Campuzano, O., Castelletti, S., Celen, C., Clauss, S., Corveleyn, A., Crotti, L., Dagradi, F., de Asmundis, C., Denjoy, I., Dittmann, S., Ellinor, P. T., Ortuño, C. G., Giustetto, C., Gourraud, J.-B., Hazeki, D., Horie, M., Ishikawa, T., Itoh, H., Kaneko, Y., Kanters, J. K., Kimoto, H., Kotta, M.-C., Krapels, I. P. C., Kurabayashi, M., Lazarte, J., Leenhardt, A., Loeys, B. L., Lundin, C., Makiyama, T., Mansourati, J., Martins, R. P., Mazzanti, A., Mörner, S., Napolitano, C., Ohkubo, K., Papadakis, M., Rudic, B., Molina, M. S., Sacher, F., Sahin, H., Sarquella-Brugada, G., Sebastiano, R., Sharma, S., Sheppard, M. N., Shimamoto, K., Shoemaker, M. B., Stallmeyer, B., Steinfurt, J., Tanaka, Y., Tester, D. J., Usuda, K., van der Zwaag, P. A., Van Dooren, S., Van Laer, L., Winbo, A., Winkel, B. G., Yamagata, K., Zumhagen, S., Volders, P. G. A., Lubitz, S. A., Antzelevitch, C., Platonov, P. G., Odening, K. E., Roden, D. M., Roberts, J. D., Skinner, J. R., Tfelt-Hansen, J., van den Berg, M. P., Olesen, M. S., Lambiase, P. D., Borggrefe, M., Hayashi, K., Rydberg, A., Nakajima, T., Yoshinaga, M., Saenen, J. B., Kääb, S., Brugada, P., Robyns, T., Giachino, D. F., Ackerman, M. J., Brugada, R., Brugada, J., Gimeno, J. R., Hasdemir, C., Guicheney, P., Priori, S. G., Schulze-Bahr, E., Makita, N., Schwartz, P. J., Shimizu, W., Aiba, T., Schott, J.-J., Redon, R., Ohno, S., Probst, V., Behr, E. R., Barc, J., & Bezzina, C. R. (2021). Enhancing rare variant interpretation in inherited arrhythmias through quantitative analysis of consortium disease cohorts and population controls. *Genetics in Medicine*, *23*, 47–58. <https://doi.org/10.1038/s41436-020-00946-5>

Wang, L., Ou, X., Sebesta, I., Vondrak, K., Krijt, J., Elleder, M., Poupetova, H., Ledvinova, J., Zeman, J., Simmonds, H. A., Tischfield, J. A., & Sahota, A. (1999). Combined adenine phosphoribosyltransferase and N-acetylgalactosamine-6-sulfate sulfatase deficiency. *Molecular Genetics and Metabolism*, *68*, 78–85. <https://doi.org/10.1006/mgme.1999.2893>

Wang, Z., Zhang, W., Wang, Y., Meng, Y., Su, L., Shi, H., & Huang, S. (2010). Mucopolysaccharidosis IVA mutations in Chinese patients: 16 novel mutations. *Journal of Human Genetics*, *55*, 534–540. <https://doi.org/10.1038/jhg.2010.65>

Whybra, C., Mengel, E., Russo, A., Bahlmann, F., Kampmann, C., Beck, M., Eich, E., & Mildenberger, E. (2012). Lysosomal storage disorder in non-immunological hydrops fetalis (NIHF): More common than assumed? Report of four cases with transient NIHF and a review of the literature. *Orphanet Journal of Rare Diseases*, *7*, 86. <https://doi.org/10.1186/1750-1172-7-86>

Xie, J., Pan, J., Guo, D., Pan, W., Li, R., Guo, C., Du, M., Jiang, W., & Guo, Y. (2019). Mutation analysis and pathogenicity identification of mucopolysaccharidosis type IVA in 8 south China families. *Gene*, *686*, 261–269. <https://doi.org/10.1016/j.gene.2018.11.051>

Xu, L., Ren, Y., Yin, J., Yang, J., Liu, Y., Zhang, J., Zhang, Y., Xiang, C., & Yang, L. (2018). Analysis of endocrine hormone metabolism level in a Chinese patient with mucopolysaccharidosis IVA: A case report. *Medicine*, *97*, e12393. <https://doi.org/10.1097/MD.0000000000012393>

Yamada, N., Fukuda, S., Tomatsu, S., Muller, V., Hopwood, J. J., Nelson, J., Kato, Z., Yamagishi, A., Sukegawa, K., Kondo, N., & Orii, T. (1998). Molecular heterogeneity in mucopolysaccharidosis IVA in Australia and Northern Ireland: Nine novel mutations including T312S, a common allele that confers a mild phenotype. *Human Mutation*, *11*, 202–208. [https://doi.org/10.1002/(SICI)1098-1004(1998)11:3<202::AID-HUMU4>3.0.CO;2-J](https://doi.org/10.1002/(SICI)1098-1004(1998)11:3%3c202::AID-HUMU4%3e3.0.CO;2-J)

Yang, C. F., Tsai, F. J., Lin, S. P., Lee, C. C., & Wu, J. Y. (2001). A novel in-frame deletion mutation (c106-111del) identified in a Taiwan Chinese patient with type IVA mucopolysaccharidosis. *Human Mutation*, *18*, 254. <https://doi.org/10.1002/humu.1187>

Yassaee, V. R., Hashemi-Gorji, F., Miryounesi, M., Rezayi, A., Ravesh, Z., Yassaee, F., & Salehpour, S. (2017). Clinical, biochemical and molecular features of Iranian families with mucopolysaccharidosis: A case series. *Clinica Chimica Acta*, *474*, 88–95. <https://doi.org/10.1016/j.cca.2017.08.017>

Ye, J., Lei, H.-l., Zhang, H.-w., Qiu, W.-j., Han, L.-s., Wang, Y., Li, X.-y., & Gu, X.-f. (2013). [Analysis of GALNS gene mutation in thirty-eight Chinese patients with mucopolysaccharidosis type IVA]. *Chinese Journal of Pediatrics/Zhonghua Er Ke Za Zhi*, *51*, 414–419.

Yubero, D., Brandi, N., Ormazabal, A., Garcia-Cazorla, À., Pérez-Dueñas, B., Campistol, J., Ribes, A., Palau, F., Artuch, R., & Armstrong, J. (2016). Targeted next generation sequencing in patients with inborn errors of metabolism. *PLoS ONE*, *11*, e0156359. <https://doi.org/10.1371/journal.pone.0156359>

Zanetti, A., D’Avanzo, F., Rigon, L., Rampazzo, A., Concolino, D., Barone, R., Volpi, N., Santoro, L., Lualdi, S., Bertola, F., Scarpa, M., & Tomanin, R. (2019). Molecular diagnosis of patients affected by mucopolysaccharidosis: A multicenter study. *European Journal of Pediatrics*, *178*, 739–753. <https://doi.org/10.1007/s00431-019-03341-8>

Zhao, H., Van Diggelen, O. P., Thoomes, R., Huijmans, J., Young, E., Mazurczak, T., & Kleijer, W. J. (1990). Prenatal diagnosis of Morquio disease type A using a simple fluorometric enzyme assay. *Prenatal Diagnosis*, *10*, 85–91. <https://doi.org/10.1002/pd.1970100204>

Zhao, Y., Meng, Y.-x., Guo, Y.-b., Du, M.-l., & Ai, Y. (2011). [Identification of a novel mutation of GALNS gene from a Chinese pedigree with mucopolysaccharidosis type IV A]. *Chinese Journal of Medical Genetics/Zhonghua Yi Xue Yi Chuan Xue Za Zhi*, *28*, 241–246. <http://doi.org/10.3760/cma.j.issn.1003-9406.2011.03.001>
